# The m6A methyltransferase METTL14 promotes oncogenic Kras induced juvenile myelomonocytic leukemia through dysregulating autophagy

**DOI:** 10.1038/s41418-025-01561-0

**Published:** 2025-08-16

**Authors:** Peihua Zhang, Keping Feng, Xiao Yu, Yi Yang, Siyu Luo, Qiao Li, Hailong Zhang, Yachun Jia, Qiaoman Fei, Xiaomin Ren, Hongwei Liu, Lin Li, Dan Yang, Gustave Munyurangabo, Jingze Yue, Qian Li, Pengyu Zhang, Lingqin Song, Aili He, Zhanping Lu, Linlin Zhang, Guangyao Kong

**Affiliations:** 1 Department of Hematology, National and Local Joint Engineering Research Center of Biodiagnosis and Biotherapy, The Second Affiliated Hospital of Xi’an Jiaotong University, Xi’an, Shaanxi China; 2 Precision Medical Institute, The Second Affiliated Hospital of Xi’an Jiaotong University, Xi’an, Shaanxi China; 3 Key Laboratory of Environment and Genes Related to Diseases, Xi’an Jiaotong University, Xi’an, Shaanxi China; 4 Department of Oncology, The Second Affiliated Hospital of Xi’an Jiaotong University, Xi’an, Shaanxi China; 5 Central Laboratory, Chongqing University Fuling Hospital, Chongqing University, Chongqing, China

**Keywords:** Cancer stem cells, Macroautophagy

## Abstract

The N6-methyladenosine (m6A) modification plays an important role in the pathogenesis of various myeloid malignancies. However, its specific role in RAS mutation-induced myeloid malignancy is incompletely understood. In this study, we found that m6A methyltransferase methyltransferase-like 14 (METTL14) was highly expressed and associated with a shorter survival in a RAS-mutation myeloid malignancy, juvenile myelomonocytic leukemia (JMML). The knockout of METTL14 was revealed to significantly promote hematopoietic stem/progenitor cells (HSPCs) expansion and suppresses disease progression in a *Kras*^*G12D/+*^ mutation-induced mouse model of JMML. Moreover, knockout of METTL14 reduces hyperproliferation of *Kras*^*G12D/+*^ HSPCs and suppresses oncogenic *Kras*^*G12D/+*^-induced myeloid disease in a cell-autonomous manner. Mechanistically, we revealed that the knockout of METTL14 reduced the autophagy levels of HSPCs by suppressing the transcription and translation of autophagy-related genes, such as autophagy-related gene 5 (*Atg5*) and autophagy-related gene 9 (*Atg9a*), through m6A modification. Furthermore, we found that the autophagy inhibition through knockout of ATG5 in *Kras*^*G12D/+*^ mutant mice promoted the expansion of HSPCs and inhibited the progression of leukemia disease, consistent with the phenotypes of knockout of METTL14. Finally, we observed that combined treatment with a m6A inhibitor and a MEK inhibitor synergistically suppressed JMML growth. Collectively, these findings highlight the critical role of METTL14 in JMML tumorigenesis and suggest that m6A modification represents a promising therapeutic target for this disease.

## Introduction

JMML is a rare, aggressive clonal hematological malignancy of early childhood, characterized by the pathological proliferation of monocytes, which frequently infiltrate organs, including the spleen (SP), liver, and lungs [1]. In its 2022 classification, the World Health Organization (WHO) categorized JMML under myeloproliferative neoplasms (MPN) [2]. Although several clinical and genetic markers have been identified, they do not fully account for the substantial clinical and biological heterogeneity observed in JMML. The disease typically exhibits rapid progression, and no standardized targeted therapy exists. Allogeneic hematopoietic stem cell transplantation (HSCT) remains the only  potentially curative treatment; however, due to the advanced stage of the disease at diagnosis and a high prevalence of comorbid conditions, most patients are initially ineligible for transplantation. Even among those who undergo HSCT, the risks of graft rejection and disease recurrence remain significant, with a reported five-year overall survival (OS) of only 52% [3].

Next-generation sequencing analyses of large JMML cohorts have revealed that approximately 90% of cases harbor driver mutations in genes associated with the Ras signaling pathway. These mutations predominantly affect genes involved in epigenetic regulation, RNA splicing, intracellular signaling, and transcriptional control. The frequently mutated gene is PTPN11, followed by NRAS, KRAS, NF1, and CBL. These mutations result in constitutive hyperactivation of the Ras-GTP-GDP loop, which can induce JMML [4–8]. Accordingly, JMML is classified as a Ras pathway-related disease syndrome. The RAS/MAPK signaling network plays a prominent role in JMML pathogenesis; however, therapeutic strategies aimed at inhibiting components of this complex signaling cascade have exhibited limited efficacy [9]. This underscores the critical need for a deeper understanding of the molecular mechanisms underlying JMML to identify novel therapeutic targets.

Previous research has established that epigenetic modifications play an important role in the pathogenesis of JMML [8]. Epigenetics refers to the stable heritable changes in biological phenotype or gene expression without nucleotide sequence changes; it involves histone covalent modification, DNA methylation modification, RNA methylation modification, genomic imprinting, gene silencing, RNA editing, and non-coding RNA [10]. Notably, m6A modification, a key epigenetic regulatory mechanism, plays a pivotal role in the regulation of stem cell function and the development of various myeloid tumors [11–15]. Since its initial discovery in the 1970s, m6A modification is the most abundant internal modification in mammalian mRNA [16]. Modification of the m6A is catalyzed by the m6A methyltransferase (writer) complex, including methyltransferase-like 3 (METTL3) and METTL14, along with their cofactor Wilms’ tumor 1-associated protein (WTAP) [17]. Within the methyltransferase family, METTL14 plays a significant auxiliary role in helping METTL3 to recognize the RNA substrates [18]. Notably, multiple studies have reported that METTL14 is implicated in the differentiation of HSPCs and the progression of myeloid disease.

For instance, METTL14 inhibits the differentiation of the HSPCs and promotes leukemogenesis through the modification of m6A in mRNA. Additionally, METTL14 is required for the development and sustenance of acute myeloid leukemia (AML) and leukemic stem/initiating cells (LSCs/LICs) [14]. Furthermore, the m6A methyltransferase complex METTL3/METTL14 promotes leukemogenesis in AML through modulation of the mdm2/p53 pathway [19]. However, the specific role of METTL14 in facilitating JMML progression remains incompletely understood, underscoring the need to elucidate its underlying function.

In this study, we demonstrate that genetic ablation of m6A methyltransferase METTL14 significantly promotes expansion of the HSPCs, thereby suppressing disease progression in JMML. Further investigation revealed that the knockout of METTL14 reduces the hyperproliferation of *Kras*^*G12D/+*^ HSPCs and suppresses oncogenic *Kras*^*G12D/+*^-induced myeloid disease in a cell-autonomous manner. Mechanistically, METTL14 suppresses the expression of autophagy-related genes through m6A modification, thereby affecting autophagic activities in HSPCs. Finally, we revealed that combined treatment with m6A and MEK inhibitor exerts a synergistic effect in inhibiting JMML progression. Overall, our findings identify METTL14 as a potential therapeutic target in JMML.

## Results

### High expression of METTL14 was associated with adverse outcomes in JMML patients, while its knockout significantly inhibited disease progression

**Fig. 1:**
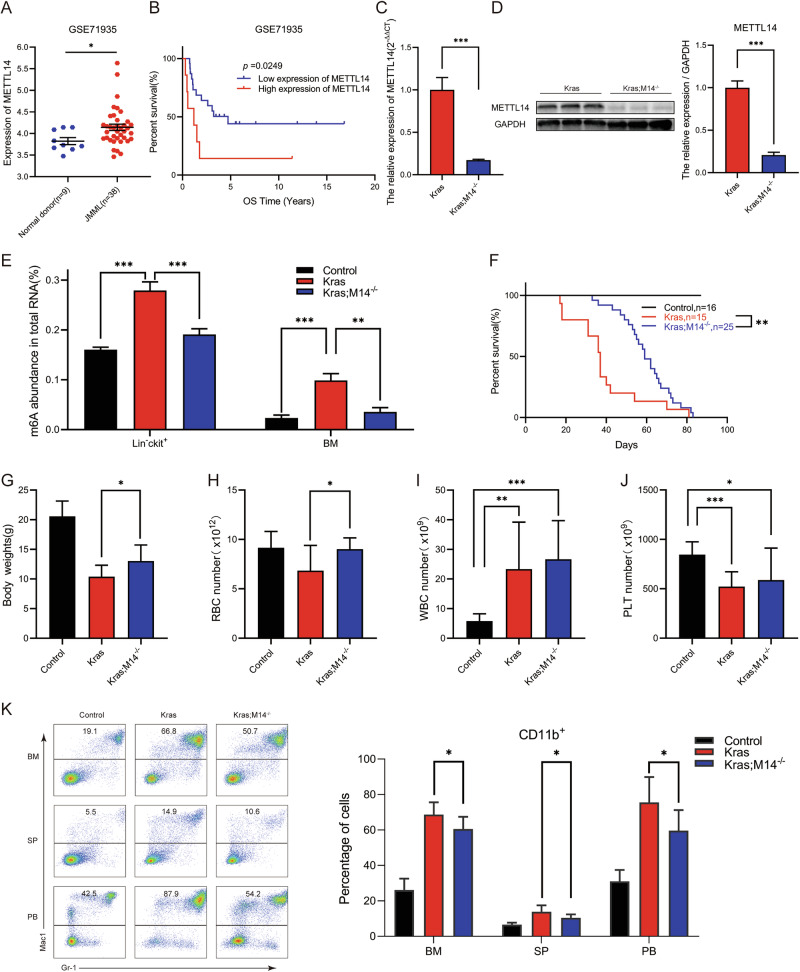
High expression of METTL14 was associated with adverse outcomes in JMML patients, while its knockout significantly inhibited disease progression. Control, Kras, and Kras;M14^-/-^ mice were sacrificed on day 1 of week seven for analysis of different hematopoietic tissues. **A** METTL14 expression in normal donors and JMML patients in GSE71935. **B** Kaplan-Meier plot. Survival curves for high (red) and low (blue) expression groups dichotomized at the optimal cutpoint are plotted and analysis of overall survival rate in the mutated cases of dataset GSE71935. **C**, **D** METTL14 mRNA expression and protein level in Kras, and Kras;M14^-/-^ mice. **E** Quantification of total RNA m6A levels in Lin^-^c-Kit^+^ cells and bone marrow cells. **F** Kaplan-Meier survival curves of different groups of mice were plotted after birth. P values were determined by the Log-rank test. **G** Quantification of body weight. **H–J** CBC analysis results. Numbers of RBC(red blood cell),WBC (white blood cell), PLT(blood platelet) are shown. **K** myeloid cell compartment in BM, SP and PB. The results are presented as means ± SD. *p < 0.05, **p < 0.01, ***p < 0.001.

To investigate the role of m6A RNA methylation in JMML, public datasets were interrogated to explore the clinical relevance of METTL3, METTL14, and WTAP for its pathogenesis. Analysis of the dataset retrieved from the GSE71935 database revealed that METTL14 expression was significantly increased in 38 patients with JMML compared with that in 9 normal donors (Fig. 1A). However, the expression levels of both METTL3 and WTAP were not significantly altered (Fig. S1A and S1C). Consequently, we assessed the prognostic value of METTL3, METTL14, and WTAP in the pathogenesis of JMML in the mutated cases obtained from the GSE71935 dataset. Our analysis revealed that elevated METTL14 or METTL3 expression was significantly associated with inferior OS in JMML patients (*p *= 0.0249, Fig. 1B, and *p* = 0.0483, Fig. S1B, respectively). While WTAP also showed a consistent trend, it did not exhibit statistical difference (*p* = 0.0904, Fig. S1D).

To determine whether METTL14 plays a significant role in the pathogenesis of *Kras*^*G12D/+*^-mediated hematopoietic malignancies, we constructed *Mettl14*^*fl/fl*^;*Mx1-Cre; Kras*^*LSL G12D/+*^ and *Mx1-Cre; Kras*^*LSL G12D/+*^ mice models. Existing literature indicates that administration of polyinosine polycytoic acid (pI-pC) in these compound mice stimulates the production of endogenous interferons (IFN), which initiates IFN-α/β-induced Cre expression from the Mx1 promoter. This processes results in oncogenic Kras expression from its endogenous locus and/or somatic loss of METTL14 [20]. In our previous study, we showed that the expression of oncogenes occurred in 67% of *Kras* ^*LSL G12D/+*^ mice at six weeks old due to leaky expression of Mx1-Cre [21]. Consequently, we leveraged the leaky expression of Mx1-Cre, precluding the need for pI-pC injection in these mice. As anticipated, pI-pC-naive mice showed significant downregulation of METTL14 (Fig. 1C, D) in 6-week-old bone marrow (BM) cells. Throughout this manuscript, we refer to wild type, *Mettl14* ^*fl/fl*^; *Mx1-Cre*; *Kras*^*LSL G12D/+*^ and *Kras*^*LSL G12D/+*^; *Mx1-Cre* mice as Control, Kras;M14^-/-^, and Kras mice, respectively.

The total m6A levels of whole bone marrow cells and the m6A levels of Lineage-negative c-Kit-positive (Lin^-^c-Kit^+^) cells were measured by sorting through magnetic beads in the JMML rodent model. The results revealed that Kras mice had significantly higher m6A levels compared with the control group in both bone marrow and Lin^-^c-Kit^+^ cells, while this increase was downregulated in the Kras;M14^-/-^ mice (Fig. 1E). Notably, the progression of leukemic disease was significantly inhibited through knockout of METTL14, thereby extending the lifespan of Kras mice (Fig. 1F). To evaluate the various hematopoietic phenotypes, the mice were euthanized on the first day of week seven after model establishment. Compared with Kras mice, Kras;M14^-/-^ mice exhibited an increase in body weight (Fig. 1G), SP mass (Fig. S1E) and index (Fig. S1F), with no significant change in liver mass (Fig. S1G). Consistent with the previous reports [22, 23], the *Kras*^*G12D/+*^ mice developed an acute MPN that closely resembled human JMML. Notably, the development of MPN was characterized by splenomegaly (Fig. S1E-1F), elevated peripheral white blood cell count, and developmental defects of both the red blood cells and megakaryocytes (Fig. 1H–J). While the knockout of METTL4 improved SP mass (Fig. S1E–1F), and anemia (Fig. 1H), there were no significant changes concerning white blood cells and platelets (Fig. 1I, J). Notably, following METTL14 knockout, the percentages of myeloid cells (CD11b^+^) were decreased in BM, SP and peripheral blood (PB), indicating suppression of *Kras*^*G12D/+*^-mediated hematopoietic malignancy (Fig. 1K).

### Knockout of METTL14 rescues *Kras*^*G12D/+*^-mediated HSPCs depletion and reduces hyperproliferation of *Kras*^*G12D/+*^ HSPCs

**Fig. 2:**
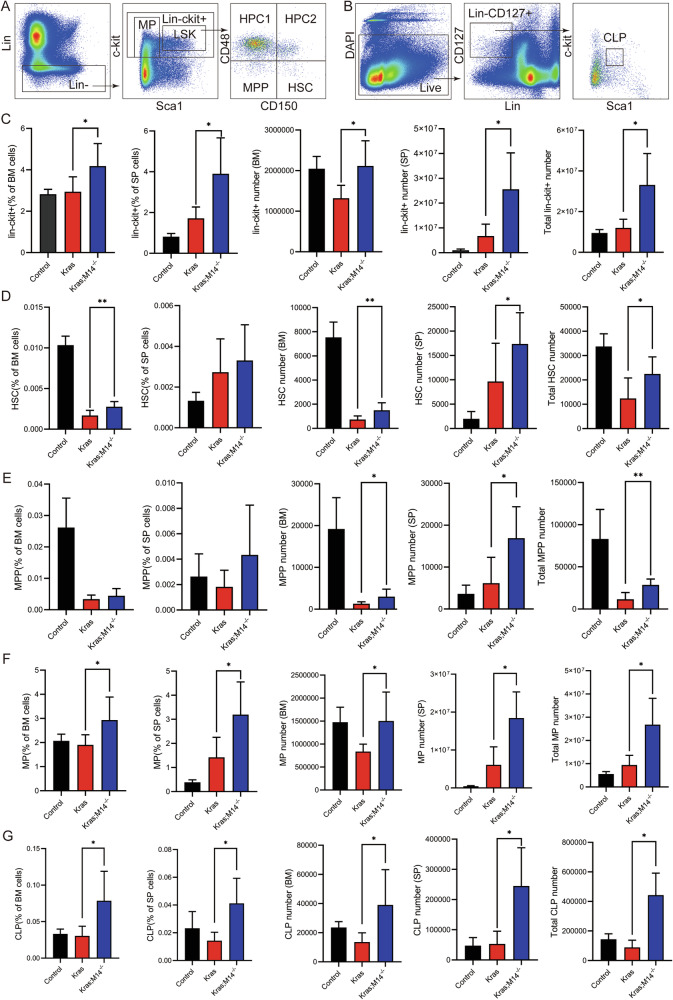
Knockout of METTL14 rescues *Kras*^*G12D/+*^-mediated HSPCs depletion. Control, Kras, and Kras;M14^-/-^ mice were sacrificed on day 1 of week seven for analysis of Lin^-^c-Kit^+^ **C**, HSCs **D**, MPPs **E**, MPs **F** and CLPs in hind limb BM (H.L.) and SP. **A** Representative example of staining and gating for HSCs, MPPs, Lin^-^c-Kit^+^cells, LSKs and MPs. **B** Representative example of staining and gating for CLPs. **C–G** The absolute Lin^-^c-Kit^+^cells, HSCs, MPPs, MPs and CLPs numbers in BM (H.L.) and SP were calculated based on BM or SP cell numbers and frequencies. Because BM (H.L.) represents 25% of whole body bone marrow, the total number of stem/progenitor cells per animal was calculated as the sum of stem/progenitor cell number in SP and four fold of stem/progenitor cell number in BM (H.L.). The results are presented as means ± SD. *P < 0.05, **P < 0.01 and ***P < 0.001.

To investigate the underlying mechanism by which METTL14 deletion suppresses myeloid disease in oncogenic Kras mice, we first examined the changes in hematopoietic stem cells (HSCs). Notably, mutant HSCs can serve as the initiating cells for JMML and chronic myelomonocytic leukemia (CMML) in mice [22, 24]. Throughout the manuscript, HSCs are defined as Lin^−^ c-Kit^+^ Sca-1^+^ CD48^−^ CD150^+^ cells (Fig. 2A). Our analysis revealed a significant increase in the frequency and quantity of Lin^−^ c-Kit^+^ cells in BM and SP of Kras;M14^-/-^ mice compared with Control and Kras mice (Fig. 2C). Additionally, we observed a notable increase in both the frequency and absolute number of Kras;M14^-/-^ HSCs in BM compared with Kras HSCs, extending to the entire body (combined BM and SP) (Fig. 2D). Consistent with existing literature [23, 24], Kras mice displayed a reduced HSCs population compared with Control mice, indicating Kras-induced oncogenic HSC depletion. However, the HSC compartment in Kras;M14^-/-^ mice was comparable to controls, suggesting that METTL14 knockout rescued the depletion of HSCs caused by Kras mutation. The total number of multipotent progenitors (MPPs, defined as Lin^−^ c-Kit^+^ Sca-1^+^ CD48^−^ CD150^−^ cells; Fig. 2A) in Kras;M14^-/-^ mice was concomitantly increased compared with that in Kras mice (Fig. 2E). Additionally, the frequency and number of the myeloid progenitors (MPs) in BM and SP were increased in Kras;M14^-/-^ mice compared with that in the Control and Kras mice (Fig. 2F). The common lymphoid progenitors (CLPs), which are defined as Lin^−^ Sca-1^low^ c-Kit^low^ CD127^+^ cells throughout the manuscript (Fig. 2B), exhibited a similar trend, specifically, the frequency and number of CLPs was increased in BM and SP in Kras;M14^-/-^ mice compared with that in the Kras mice (Fig. 2G).

Further analysis focused on the quantification of the MPs in all groups (Fig. S2A–2D). Throughout this manuscript, common myeloid progenitors (CMPs) are defined as Lin^−^ Sca-1^−^ c-Kit^+^ CD34^+^ CD16/32^low^ cells; granulocyte-macrophage progenitors (GMPs) are defined as Lin^−^Sca-1^−^c-Kit^+^ CD34^+^ CD16/32^+^ cells; and megakaryocyte-erythroid progenitors (MEPs) are defined as Lin^−^ Sca-1^−^ c-Kit^+^ CD34^−^ CD16/32^−^ cells (Fig. S2A). In Kras;M14^-/-^ mice, the number of MPs, including CMPs, GMPs was significantly increased in BM and SP compared with that in Kras mice (Fig. S2B and S2C); in contrast, the number of MEPs showed no significant difference compared with the Control mice (Fig. S2D). Collectively, our findings indicate that knockout of METTL14 suppressed myeloid diseases in Kras mice by rescuing *Kras*^*G12D/+*^-mediated HSC depletion.

**Fig. 3:**
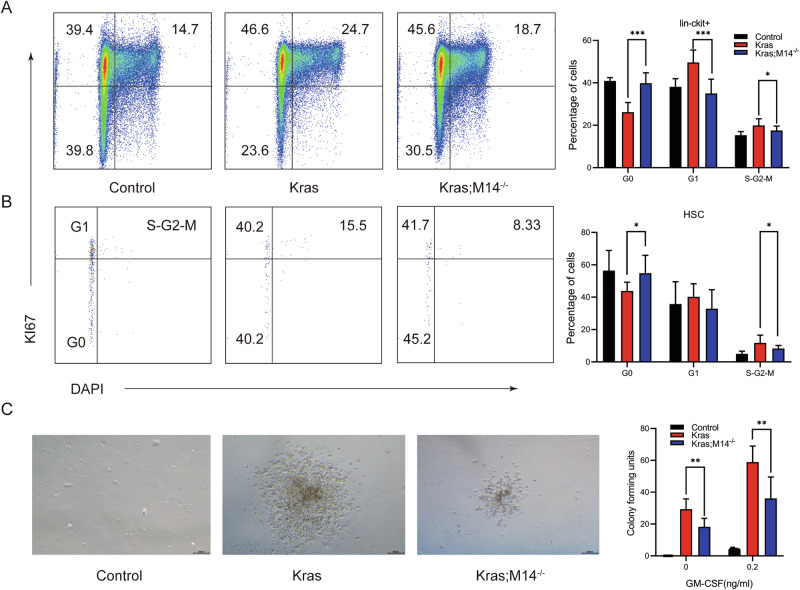
Loss of METTL14 reduces hyperproliferation of *Kras*^*G12D/ +*^ HSPCs. Control, Kras, and Kras;M14^-/-^ mice were sacrificed on day 1 of week seven for Cell cycle analysis of Lin^-^c-Kit^+^ and HSCs in BM. 5 × 10^4^ BM cells from different groups of mice were plated in methylcellulose-based medium M3234 with or without GM-CSF. **A, B** Representative example of staining and gating for G0, G1 and S/G2/M phase. Cell cycle analysis of bone marrow Lin^-^c-Kit^+^ cells **A** and HSCs **B** using Ki67 and DAPI. **C** Quantification of colonies cultured for 7 days in methylcellulose-based medium M3234. The results are presented as mean ± SD. *P < 0.05; **P < 0.01; ***P < 0.001.

Cell cycle analysis revealed that, compared with the Control group, Kras Lin^−^c-Kit^+^ cells and HSCs exhibited significant hyperproliferation. However, this hyperproliferative phenotype was significantly reduced in the Kras;M14^-/-^ Lin^−^c-Kit^+^ cells and HSCs (Fig. 3A, B). These observations were consistent with our previous report [21]. Moreover, consistent with our cell cycle analysis, Kras;M14^-/-^ mice BM cells formed significantly less colonies compared with that of the Kras mice BM cells in the presence of 0.2 ng/ml of GM-CSF (Fig. 3C). In addition, we analyzed the HSPCs in 8-week-old mice with Kras and Kras;M14^-/-^ genotypes. Our findings revealed that the alterations in each cell subset mirrored those observed in 6-week-old mice (Fig. S3A–S3M). Notably, the changes in CD11b^+^ cell populations in BM, SP and PB were also in line with these observations (Fig. S3N). Additionally, cell cycle analysis further corroborated the experimental results from 6-week-old mice (Fig. S3O and S3P). These findings indicate the stability of phenotypic changes across different cell types at distinct time points. Collectively, our findings suggest that knockout of METTL14 reduces hyperproliferation of Kras HSPCs.

### Knockout of METTL14 suppresses oncogenic *Kras*^*G12D/+*^-induced myeloid disease in a cell-autonomous manner

**Fig. 4:**
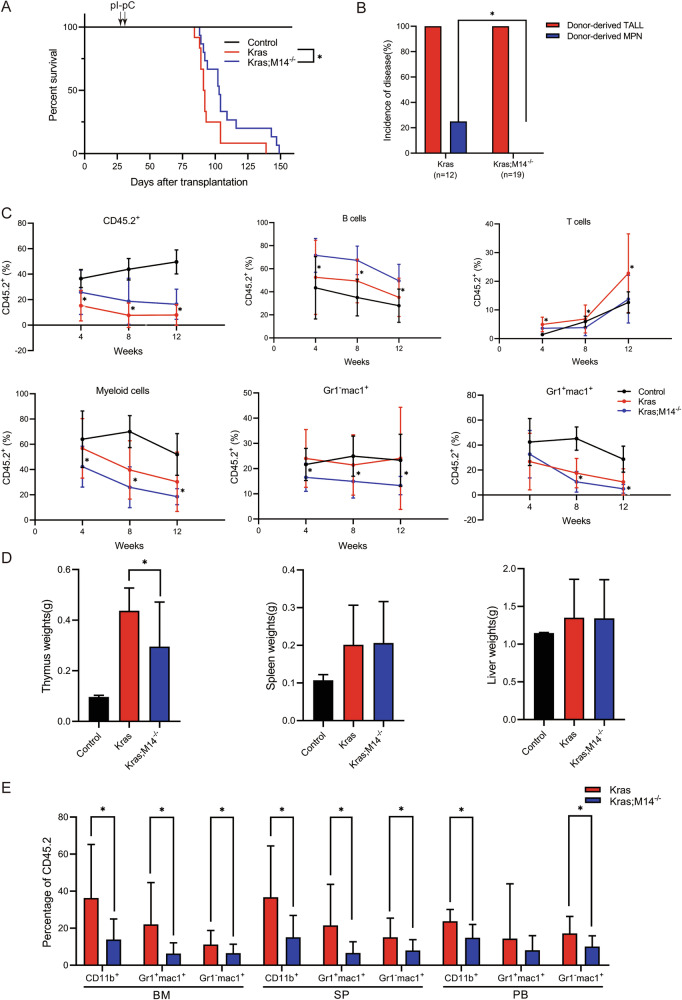
Knockout of METTL14 suppresses oncogenic *Kras*^*G12D/+*^-induced myeloid disease in a cell-autonomous manner. Control, Kras, and Kras;M14^-/-^ mice were sacrificed on day 1 of week seven for transplantation experiments. Lethally irradiated mice were transplanted with 2.5×10^5^ BM cells from different groups of mice along with same number of competitor cells. Four weeks after transplantation, Mx1-Cre expression was induced by pI-pC injections. **A** Kaplan-Meier survival curves of different groups of recipient mice were plotted against days after transplantation. *P* values were determined by the Log-rank test. **B** Disease distribution patterns in recipient mice transplanted with Kras or Kras;M14^-/-^ cells. We define the mice with a myeloid disease when donor-derived monocytes consist >20% of white blood cells in peripheral blood. Chi-square analysis was performed. **C** Total donor-derived cells and donor-derived T cells, B cells, myeloid cells, neutrophils and monocytes(CD45.2^+^) in different groups of recipients were evaluated regularly after transplantation. Of note, 4-week data were collected right before pI-pC injections. **D** Quantitative analysis of Thymus weight, spleen weight and Liver weight. **E** Quantitative analysis of donor-derived myeloid cells, monocytes and neutrophils in BM, SP and PB from moribund recipient mice. The results are presented as mean ± SD. *P < 0.05; **P < 0.01; ***P < 0.001.

An equivalent number of Control, *Kras*^*G12D/+*^; *Mx1-Cre*, or *Kras*^*G12D/+*^; *Mettl14* ^*fl/fl*^; *Mx1-Cre* bone marrow cells (CD45.2^+^) along with congeneic competitor cells (CD45.1^+^) were transplanted into lethally irradiated mice to determine whether METTL14 deficiency plays a cell-autonomous role in inhibiting leukemogenesis in the Kras model. Four weeks after transplantation, recipient mice were injected with pI-pC to induce expression of oncogenic *Kras* ^*G12D/+*^ and deletion of METTL14. The results revealed that Kras;M14^-/-^ cell recipients had extended lifespan compared with the Kras cell recipients (Fig. 4A). In addition, we found that approximately 20% of the recipient mice transplanted with Kras cells developed a donor-derived MPN, whereas none of recipients transplanted with Kras;M14^-/-^ cells developed MPN (Fig. 4B). Donor derived MPN was defined as previously described [25]: donor-derived CD45.2^+^ cells constitute >50% in the peripheral blood of recipients and >20% of donor-derived cells were Gr1^−^Mac1^+^ monocytes.

The proportion of total donor-derived cells and donor-derived B cells in PB of Kras;M14^-/-^ recipients was significantly increased (Fig. 4C). In contrast, the proportion of donor-derived T cells and Mac1^+^ myeloid cells (including Gr1^−^Mac1^+^ monocytes and Gr1^+^Mac1^+^ neutrophils) in PB of Kras;M14^-/-^ recipient mice was significantly reduced (Fig. 4C), potentially resulting in the extended OS time of the Kras;M14^-/-^ recipient mice. All the recipients of Kras;M14^-/-^ cells and Kras cells developed T-ALL (Fig. 4B). We captured the moribund recipients for analysis, with the results indicating that the thymus mass of Kras;M14^-/-^ cell-derived recipients was significantly reduced compared with that of the Kras cell recipients, whereas the SP weights and liver weights were comparable to those of Kras cell recipients (Fig. 4D). Compared with the T-ALL that developed in recipients of Kras;M14^-/-^ cells, Kras derived tumors contained a significant percentage of CD4^−^CD8^−^ T-cells, including DN1 (CD44^+^CD25^-^) and DN2 (CD44^+^CD25^+^) T-cells (Fig. S4A), indicating the development of a significantly immature phenotype. In addition, donor-derived myeloid cells, monocytes, and neutrophils from moribund recipient mice were significantly reduced in BM, SP and PB in recipients of Kras;M14^-/-^ cells compared with recipients of Kras cells (Fig. 4E). Collectively, our findings demonstrate that knockout of METTL14 inhibits both T-ALL and MPN development in Kras mice in a cell-autonomous manner.

### Knockout of METTL14 restraints autophagy by targeting *Atg5* and *Atg9a* via m6A modification in Kras HSPCs

**Fig. 5:**
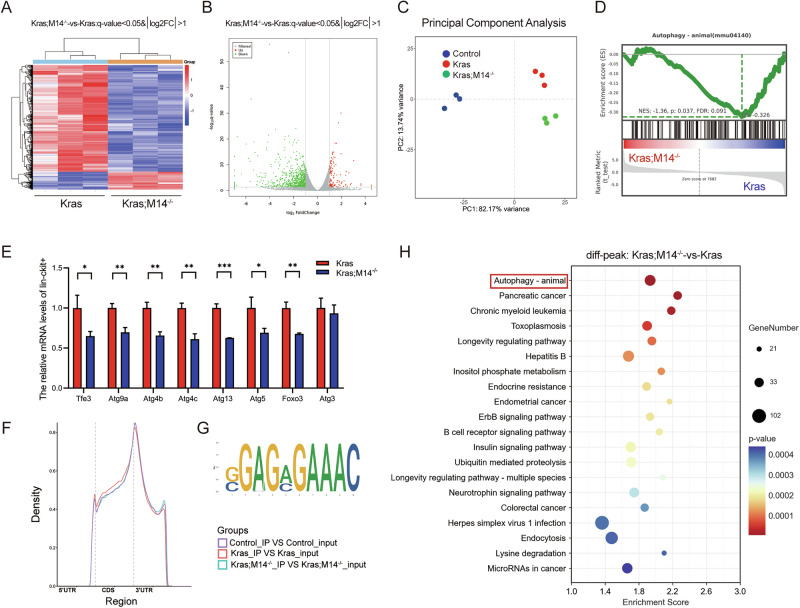
Knockout of METTL14 regulateds autophagy in Kras HSPCs. Control, Kras, and Kras;M14^-/-^ mice were sacrificed on day 1 of week seven and HSPCs (Lin^-^ckit^+^ cells) were separated from the BM by magnetic bead sorting for RNA sequencing and micro m6A sequencing. **A, B** Heatmap and Volcano Plot of RNA-seq analysis of BM-derived Lin^-^c-Kit^+^ cells of Kras and Kras;M14^-/-^ mice. **C** Principol Component Analysis of BM-derived Lin^-^c-Kit^+^ cells of Kras and Kras;M14^-/-^ mice. **D** GSEA analysis of differentially expressed genes in Kras and Kras;M14^-/-^ mice BM-derived Lin^-^c-Kit^+^ cells on RNA -seq. **E** The expression of Autophagy genes was verified by qRT-PCR. **F** The density distribution of total m6A peaks in the BM-derived Lin^-^c-Kit^+^ cells of Kras and Kras;M14^-/-^ mice. **G** Predominant consensus motif AAAC was detected in Lin^-^c-Kit^+^ BM cells. **H** KEGG analysis of the genes with significantly changed in Kras and Kras;M14^-/-^ mice BM-derived Lin^-^c-Kit^+^ cells on m6A -seq.The results are presented as mean ± SD. *P < 0.05; **P < 0.01; ***P < 0.001.

**Fig. 6:**
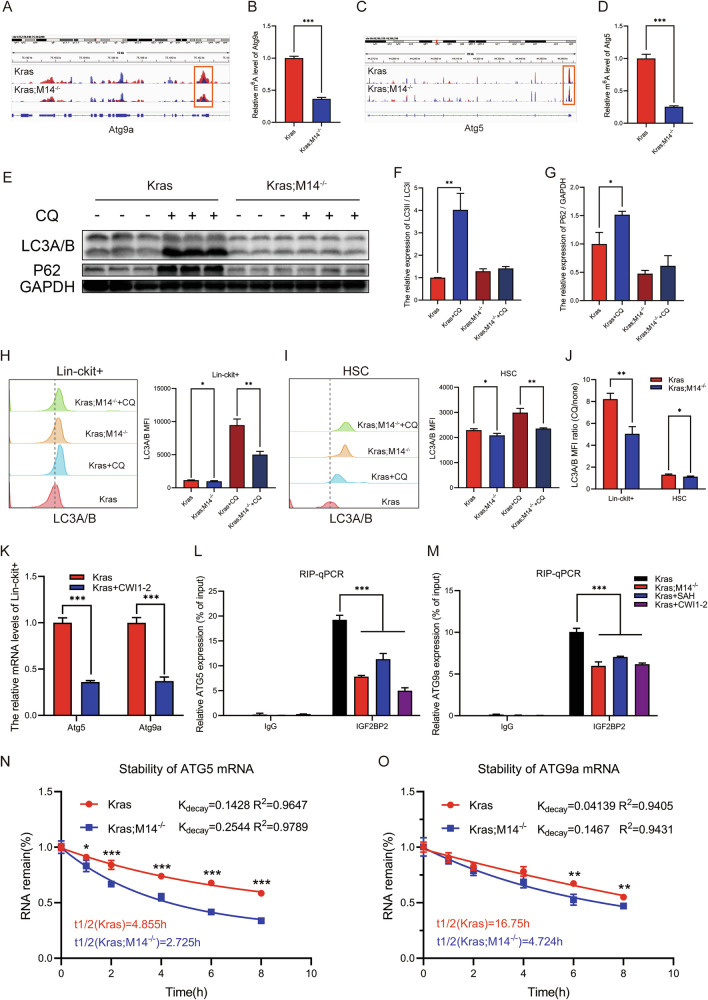
Knockout of METTL14 restraints autophagy by targeting *Atg5* and *Atg9a* via m6A modification in Kras HSPCs. **A** Integrative Genomics Viewer (IGV) tracks display m^6^A abundance in *Atg9a* transcripts in Kras and Kras;M14^-/-^ mice. **B** MeRIP-qPCR analysis m^6^A enrichment of *Atg9a* transcripts in Kras Lin^-^c-Kit^+^ cells and Kras;M14^-/-^ mice Lin^-^c-Kit^+^ cells. **C** Integrative Genomics Viewer (IGV) tracks display m^6^A abundance in *Atg5* transcripts in Kras and Kras;M14^-/-^ mice. **D** MeRIP-qPCR analysis m^6^A enrichment of *Atg5* transcripts in Kras Lin^-^c-Kit^+^ cells and Kras;M14^-/-^ Lin^-^c-Kit^+^ cells. **E–G** Western blotting analysis of LC3 and p62 protein levels in BM cells derived from Kras and Kras;M14^-/-^ mice. **H** Representative LC3 flow cytometry histograms and LC3 MFI quantification of Lin^-^c-Kit^+^ cells derived from Kras and Kras;M14^-/-^ mice. **I** Representative LC3 flow cytometry histograms and LC3 MFI quantification of HSCs derived from Kras and Kras;M14^-/-^ mice. **J** Quantification of median fluorescent intensity (MFI) of LC3 in Lin^-^c-Kit^+^ cells and HSCs with and without chloroquine (CQ) treatment. **K** The expression of Atg5 and Atg9a were verified by qRT-PCR. **L** Real-time PCR analysis of RIP assays in BM cells showing the direct binding between the IGF2BP2 protein and Atg5 mRNA. **M** Real-time PCR analysis of RIP assays in BM cells showing the direct binding between the IGF2BP2 protein and Atg9a mRNA. **N** mRNA half-life (t1/2) of Atg5 after actinomycin D treatment for 0,1,2,4,6,and 8 h in Kras and Kras; M14^-/-^ mice. **O** mRNA half-life (t1/2) of Atg9a after actinomycin D treatment for 0,1,2,4,6,and 8 h in Kras and Kras;M14^-/-^ mice. The results are presented as mean ± SD. *P < 0.05; **P < 0.01; ***P < 0.001.

To determine the mechanism of HSPCs expansion and inhibition of leukemic disease mechanisms in Kras;M14^-/-^ mice, we conducted RNA sequencing (RNA-Seq) analysis of HSPCs (Lin^-^c-Kit^+^ cells) sorted via magnetic beads in each group of mice to identify the METTL14 targets. The RNA sequencing results revealed that 859 genes were differentially expressed after METTL14 knockout, with 736 downregulated while 123 upregulated (Fig. 5A, B). Principal component analysis (PCA) showed that the samples in each group exhibited good repeatability (Fig. 5C). Notably, GSEA analysis revealed significant enrichment of the autophagy-related pathway (Fig. 5D). Macroautophagy, also known as as autophagy, is a cellular recycling mechanism whereby autophagosomes wrap around intracellular substances and fuse with lysosomes to degrade the phagocytic cellular components [26]. We hypothesized that dysregulated autophagy potentially results in HSPCs expansion and subsequent inhibition of leukemic disease processes in Kras;M14^-/-^ mice. Consequently, we conducted qRT-PCR with the results revealing that most autophagy-related genes such as *Atg5*, *Atg9a*, *Atg4b*, *Atg4c* and *Atg13* were significantly downregulated in Kras;M14^-/-^ mice HSPCs (Fig. 5E). Autophagy is significantly implicated in the maintenance of quiescence and stemness in HSC [27, 28]. Previous research has demonstrated that m6A modification plays a crucial role in regulating autophagy [29, 30]. To further explore how METTL14 knockout inhibits the leukemic disease process, we conducted methylated RNA immunoprecipitation sequencing (MeRIP-seq) analysis of HSPCs sorted through magnetic beads in each group of mice to identify the METTL14 targets. The analysis revealed that m6A peaks were distributed in the 3’-untranslated (3’-UTR) and the coding region (CDS). Compared with the Kras group, the CDS m6A level was significantly decreased after METTL14 knockout, with levels nearly comparable to those of the Control group (Fig. 5F). As shown in Fig. 5G, the consensus motif GGAC was abundant within m6A sites in both the Kras and Kras;M14^-/-^ mice HSPCs. Similarly, KEGG analysis of micro M6A-Seq revealed enrichment of autophagy-related pathways (Fig. 5H), consistent with previous findings from RNA-seq analysis. Additionally, MeRIP-qPCR analysis indicated that knockout of METTL14 significantly decreased the m6A modification levels of *Atg9a* and *Atg5* mRNA (Fig. 6A–D), consistent with m6A-seq results. Western blotting was performed with the result revealing a reduction in the rate of autophagic activities in whole bone marrow cells (Fig. 6E–G) after knockout of METTL14; notably, this method was conducted by detecting the autophagosomal marker microtubule-associated protein 1 A/1B light chain 3B (LC3) protein [31] and the cargo protein marker p62 [also referred to as sequestosome 1 (SQSTM1)] [32]. Importantly, these observations were consistent with the m6A-seq analysis. To further examine the levels of autophagy in HSPCs, autophagy levels were quantified using intracellular flow cytometry by detecting the autophagomal marker LC3 protein, along with the cell surface panel for HSPCs. We found that inhibition of autophagy with chloroquine phosphate significantly reduced LC3 staining in the Lin^-^c-Kit^+^ cells of Kras;M14^-/-^ mice (Fig. 6H, J), with consistent results in HSC (Fig. 6I, J), suggesting reduced cell-specific autophagy in the HSPCs after knockout of METTL14. Our previous results showed that the levels of *Atg5* and *Atg9a* m6A were significantly decreased, with the mRNA level also decreased following METTL14 knockout in Kras mice (Fig. 6A–D, Fig. 5E). Research has identified a novel class of m6A reader proteins, the insulin-like growth factor 2 mRNA-binding protein (IGF2BP) family, which stabilizes m6A-containing mRNAs and promotes their translation through their K homology domains [33]. In this study, we revealed that mRNA of *Atg5* and *Atg9a* was reduced after treatment of Kras mice cells with IGF2BP2 inhibitor CWI1-2 (Fig. 6K). Notably, CWI1-2 has been shown to bind directly to IGF2BP2 to competitively inhibit its binding to RNA targets [34]. Meanwhile, RIP-qPCR analysis indicated that the mRNA of *Atg5* and *Atg9a* were increased by ten-fold or higher after treatment with the IGF2BP2 antibody in Kras mice cells compared with IgG antibody, with the increase significantly decreased after knockout of METTL14 or treatment with m6A inhibitor, SAH or IGF2BP2 inhibitor, CWI1-2 (Fig. 6L, M). These observations confirmed that m6A modulators regulated *Atg5* and *Atg9a* expression. We also found that the half-life of *Atg5* and *Atg9a* mRNA were significantly shortened in METTL14 knockout Kras mice cells (*Atg5*: 4.855 h versus 2.725 h, *Atg9a*: 16.75 h versus 4.724 h, respectively; Fig. 6N, O), which suggested that m6A modification impedes the degradation of autophagy-regulating genes *Atg5* and *Atg9a* mRNA. Collectively, these findings establish a direct role of METTL14-mediated m6A in maintaining *Atg5* and *Atg9a* expression. Consequently, our results suggest that the knockout of METTL14 can promote the expansion of HSPCs and inhibit the disease process of leukemia by inhibiting HSPCs autophagy.

### Inhibition of autophagy via knockout of ATG5 promotes HSPCs amplification and suppresses the leukemic disease process

**Fig. 7:**
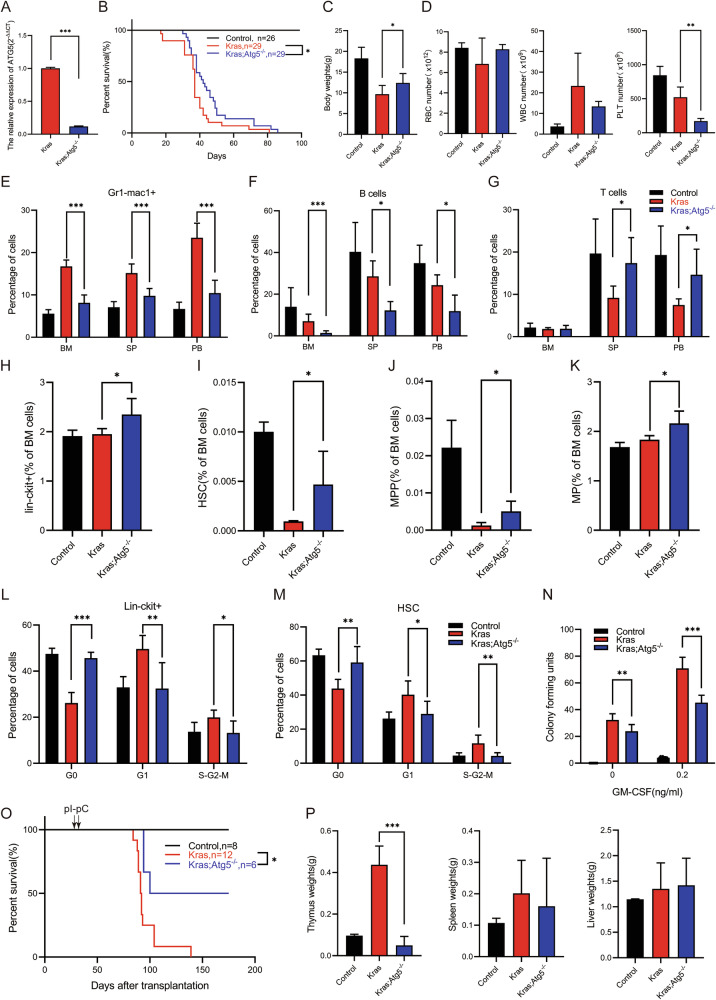
Inhibition of autophagy via knockout of ATG5 promotes HSPCs amplification and suppresses the leukemic disease process. **A** Control, Kras, and Kras;Atg5^-/-^ mice were sacrificed on day 1 of week seven for analysis of different hematopoietic tissues. **A** *Atg5* mRNA expression level in Kras, and Kras;Atg5^-/-^ mice. **B** Kaplan-Meier survival curves of different groups of mice were plotted after birth. *P* values were determined by the Log-rank test. **C** Quantification of body weight. **D** CBC analysis results. Numbers of RBC(red blood cell),WBC (white blood cell), PLT(blood platelet) are shown. **E** Monocytes (Gr1^-^CD11b^+^) compartment in BM, SP and PB. **F** B cells compartment in BM, SP and PB. **G** T cells compartment in BM, SP and PB. **H–K** The percentage of Lin^-^c-Kit^+^ cells,HSCs, MPPs and MPs in BM. **L** Cell cycle analysis of BM lineage-negative ckit-positive cells(Lin^-^c-Kit^+^ cells) using Ki67 and DAPI. **M** Cell cycle analysis of BM HSCs using Ki67 and DAPI. **N** Quantification of colonies cultured for 7 days in methylcellulose-based medium M3234. **O** Kaplan-Meier survival curves of different groups of recipient mice were plotted against days after transplantation. *P* values were determined by the Log-rank test. **P** Quantitative analysis of Thymus weight, spleen weight and Liver weight. The results are presented as mean ± SD. *P < 0.05; **P < 0.01; ***P < 0.001.

We hypothesized that METTL14 knockout significantly reduced the methylation levels of mRNA of autophagy-related genes, to suppress their expression. Inhibition autophagy further promoted HSPCs expansion and inhibited leukemic progression. We also explored whether there were associations between autophagy inhibition and METTL14 knockout in *Kras*^*G12D/+*^ mice. Notably, *Atg5* spans the 6q21 region of chromosome 6 with a size of 141.34 kb and 10 exons, and primarily encodes a 275-amino-acid autophagy protein 5 [35]. Due to stress, the autophagy process is initiated via activation of the *Atg* genes, which encode proteins responsible for autophagy-related responses localized to sites of autophagosome formation [36]. Among these proteins, ATG5 is a key protein in regulating autophagosome formation–which is the most critical step in the autophagy process. Given the critical role of *Atg5* in autophagy, we generated *Atg5*^*fl/fl*^;*Mx1-Cre*; *Kras*^*LSL G12D/+*^ mice model with the capability of inhibiting autophagy to determine whether autophagy plays an important role in *Kras*^*G12D/+*^-mediated hematopoietic malignancies. Based on the previous findings, we leveraged leaky expression of Mx1-Cre, precluding the need for pI-pC injection. Throughout this article, we refer to wild type, *Atg5*^*fl/fl*^;*Mx1-Cre*;*Kras*^*LSL G12D/+*^ and *Kras*^*LSL G12D/+*^;*Mx1-Cre* mice as Control, Kras;Atg5^-/-^ and Kras mice, respectively. The knockout efficiency were examined in Lin^-^c-Kit^+^ cells using the qRT-PCR technique (Fig. 7A).

The knockout of ATG5 significantly inhibited the leukemic disease process and extended the lifespan of Kras mice, consistent with the knockout of METTL14 results (Fig. 7B). Control, Kras, and Kras;Atg5^-/-^ mice were euthanized on the first day of week seven, following the model establishment, to analysis of different hematopoietic phenotypes (Fig. 7C and Fig. S5A-5B). Compared with Kras mice, Kras;Atg5^-/-^ mice showed increased body weight (Fig. 7C) and spleen mass (Fig. S5A), however, liver mass did not change significantly (Fig. S5B), which was consistent with the observation in Kras;M14^-/-^ mice. The CBC showed thrombocytopenia, with no significant changes in white and red blood cells population (Fig. 7D). Consistent with METTL14 knockout, the number of monocytes (Gr1^-^CD11b^+^) was significantly reduced in the BM, SP, and PB after ATG5 knockout (Fig. 7E), indicating that inhibition of autophagy suppressed *Kras*^*G12D/+*^-mediated hematopoietic malignancies. Notably, following ATG5 knockout, B cells decreased in the BM, SP and PB(Fig. 7F), while T cells increased in the SP and PB, but no change was observed in BM (Fig. 7G), indicating that the inhibition of autophagy may have an impact on the development of lymphocytes. Then we wondered how the knockout of ATG5 in Kras mice inhibited the process of JMML. Additionally, flow cytometry was used to quantify HPSCs using the cell surface panel (as previously described). We found that in BM, the frequency of Lin^−^c-Kit^+^ cells in Kras;Atg5^-/-^ mice was significantly higher than that in Kras mice alone (Fig. 7H), which was consistent with findings involving the knockout of METTL14. We also found that the frequency of Kras;Atg5^-/-^ HSCs was significantly increased in the BM compared with Kras HSCs (Fig. 7I), with the same trend observed in HPC1 (Fig. S5C), HPC2 (Fig. S5D), MPP (Fig. 7J), MP (Fig. 7K), and GMP (Fig. S5F); however, CMP exhibited an inverse trend (Fig. S5E), whereas the MEPs were comparable to the Controls (Fig. S5G). Notably, these observation were consistent with the changes in HSPCs following METTL14 knockout, suggesting that METTL14 knockout inhibits autophagy by reducing the methylation level of autophagy-associated genes, which ultimately inhibits myeloid disease in *Kras*^*G12D/+*^ mice.

Cell cycle analysis, which was consistent with the previous results, demonstrated that Kras Lin^−^c-Kit^+^ cells and HSCs were significantly proliferative compared with those in the Controls; however, the hyperproliferative phenotype of Kras;Atg5^-/-^ Lin^−^c-Kit^+^ cells and HSCs was significantly reduced (Fig. 7L, M). Notably, Kras;Atg5^-/-^ mice BM cells formed significantly fewer colonies than Kras mice BM cells in the presence of 0.2 ng/ml of GM-CSF, consistent with our cell cycle analysis (Fig. 7N). Collectively, our findings demonstrate that knockout of ATG5 reduces hyperproliferation of *Kras*^*G12D/+*^ HSPCs, which is consistent with knockout of METTL14. To determine whether ATG5 deficiency plays a cell-autonomous role in inhibiting leukemogenesis in *Kras*^*G12D/+*^ model, we transplanted an equal population of Control, *Kras*^*G12D/+*^; *Mx1-Cre*, or *Kras*^*G12D/+*^; *Atg5*^*fl/fl*^; *Mx1-Cre* BM cells (CD45.2^+^) along with congeneic competitor cells (CD45.1^+^) into lethally irradiated mice. Four weeks after transplantation, recipient mice were injected with pI-pC to induce expression of oncogenic Kras^*G12D/+*^ and deletion of ATG5. The results indicated that Kras;Atg5^−/−^ cell recipients exhibited extended survival time compared with the Kras cell recipients (Fig. 7O). Analysis of the moribund recipients revealed that the thymus mass of Kras;Atg5^−/−^ cell-derived recipients was significantly reduced compared with that of the Kras cell recipients, whereas the spleen and liver weights were comparable to those of Kras cell recipients (Fig. 7P), suggesting a less severe T-ALL. Collectively, our results indicate that knockout of ATG5 inhibits T-ALL development in Kras mice in a cell-autonomous manner, which is consistent with the observations obtained following the METTL14 knockout.

### Combined MEK inhibitor and m6A inhibitor treatment effectively suppresses the growth of JMML cells

**Fig. 8:**
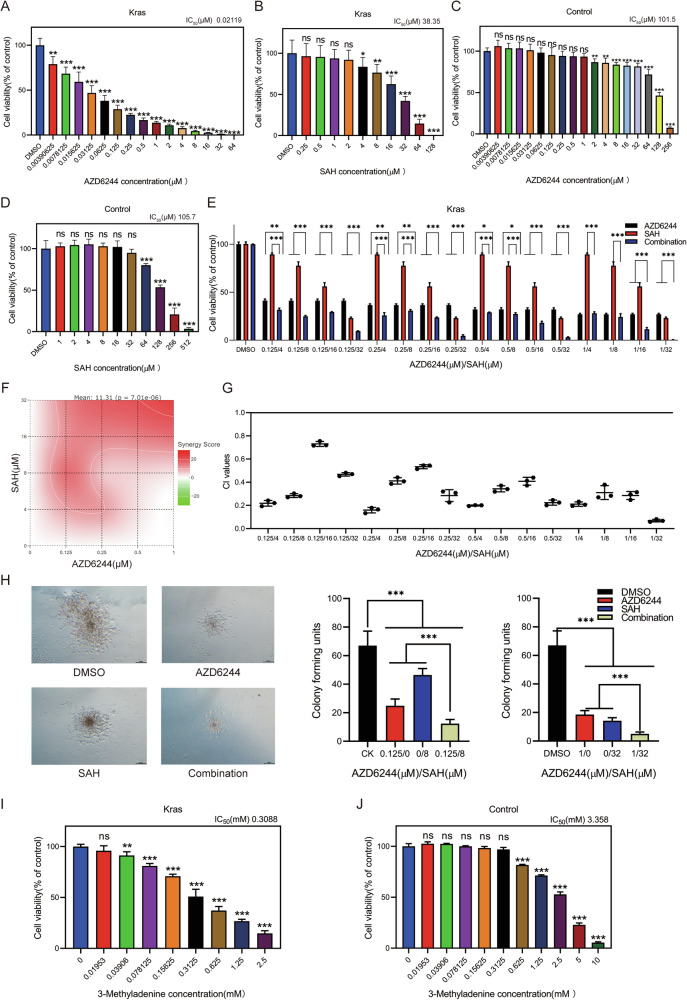
Combined MEK inhibitor and m6A inhibitor treatment effectively suppresses the growth of JMML cells. Combined AZD6244 and SAH treatment effectively inhibits the growth of mouse leukemia cells in vitro. Leukemia cells from moribund Kras mice were cultured in 96-well plates in the presence of vehicle or various concentrations of AZD6244 and/or SAH for 5 days. **A** Cell viability of Kras mice BM cells treated with AZD6244 for 5 days. **B** Cell viability of Kras mice BM cells treated with SAH for 5 days. **C** Cell viability of Control mice BM cells treated with AZD6244 for 5 days. **D** Cell viability of Control mice BM cells treated with SAH for 5 days. **E** Cell viability of Kras mice BM cells treated with AZD6244 and SAH, alone or in combination for 5 days. **F** Synergistic effect of AZD6244 with SAH on inhibition of the survival/growth of Kras mice BM cells as determined by the HSA independent model. Mean score represents the percentage of response beyond expectation due to drug interactions. **G** CI values of Kras mice BM cells treated for 5 days with AZD6244 and SAH, alone or in combination, were calculated using CompuSyn. The index value less than 1 indicates synergism. **H** Quantification of colonies cultured with AZD6244 and SAH, alone or in combination for 7 days in methylcellulose-based medium M3234. **I** Cell viability of Kras mice BM cells treated with 3-Methyladenine for 24 h. **J** Cell viability of Control mice BM cells treated with 3-Methyladenine for 24 h. Cell number was quantified using the Cell Counting Kit-8 assay. The results are presented as mean ± SD. *P < 0.05; **P < 0.01; ***P < 0.001.

To determine whether inhibition of MEK and/or m6A modification effectively controls oncogenic Kras cell growth, we isolated BM cells from moribund Kras mice. We then cultured them in the absence or presence of AZD6244 (a MEK inhibitor [37, 38]) and/or SAH (an inhibitor for METTL3-METTL14 heterodimer complex (METTL3-14) [39]). The results demonstrated that either AZD6244 or SAH significantly reduced the viability of Kras cells in a dose-dependent manner (Fig. 8A, B); however, there was no significant effect on the Control cells despite effective drug concentration (Fig. 8C, D). Although Kras cells demonstrated variable sensitivity to independent administration of either AZD6244 or SAH, they exhibited elevated sensitivity when the two treatments were employed in combination (Fig. 8E). We found that the combination of AZD6244 and SAH synergistically abrogated the growth of Kras cells (Fig. 8E). Notably, the combination indices exhibited values < 1 (Fig. 8F, G), indicating a synergistic effect between AZD6244 and SAH. Meanwhile, the combination of AZD6244 and SAH significantly resulted in fewer colony formation of BM cells in Kras mice compared with the independent administration of either AZD6244 or SAH in the presence of 0.2 ng/ml of GM-CSF. However, the application of independent treatment also differentially reduced colony formation in Kras mice (Fig. 8H). Considering the pivotal role of elevated autophagy in JMML occurrence, pharmaceuticals agents targeting the autophagic pathway may exhibit significant therapeutic potential in the treatment of JMML. We isolated BM cells from moribund Kras mice and cultured them in the absence or presence of 3-Methyladenine (an autophagy inhibitor). As anticipated, our results showed that 3-Methyladenine significantly reduced the viability of Kras cells in a dose-dependent manner (Fig. 8I), however, its effective drug concentration had no significant effect on the Control cells (Fig. 8J). Our findings demonstrate that the combined inhibition of MEK and METTL14 synergistically reduces the growth of Kras mutant JMML cells.

## Discussion

In this study, we have demonstrated that the downregulation of METTL14 promotes HSPCs expansion and inhibits the leukemic disease process. Moreover, downregulation of METTL14 in Kras hematopoietic cells inhibits MPN in a cell-autonomous manner. Furthermore, METTL14 deficiency suppresses myeloid disease by inhibiting autophagy in *Kras*^*G12D/+*^ HSPCs.

Our study identified an oncogenic function of METTL14 in *Kras*^*G12D/+*^-induced MPN. This finding was consistent with METTL14 gene function in AML and LSCs/LICs [14]. In humans, METTL3–METTL14-mediated m6A was found to promote the development of acute myeloid leukemia and maintain leukemia-initiating cells [12–14].

Previous studies have shown that expressing mutant *Kras*^*G12D/+*^ protein in the endogenous murine locus rapidly induces a fatal myeloproliferative disorder. This disorder is characterized by 100% penetrance due to HSC depletion and hyperproliferation. Notably, HSC hyperproliferation tends to be associated with reduced HSC self-renewal and subsequent depletion caused by self-exhaustion. Although, there was no significant change in the frequency of BM HSCs after knockout of METTL14 in normal mice [40], knockout of METTL14 can rescue *Kras*^*G12D/+*^-induced HSC depletion and hyperproliferation, which is an observation potentially attributed to the increased self-renewal of HSPCs after knockout of METTL14 (Fig. S6A). Notably, m6A levels of HSCs were higher compared with those of downstream progenitor cell populations, including LSK, CMP, GMP, and MEP (Fig. S6D). This observation suggests that METTL14 may affect differentiation at the HSC stage in the context of JMML. In addition, we found that METTL14 is involved in the activation of MEK/ERK signaling induced by mutant Kras, however, the expression of METTL14 is not caused by mutant Kras-induced MEK/ERK activation (Fig. S6E–6H). These findings suggest that METTL14 plays a crucial role in *Kras*^*G12D/+*^-mediated myeloid neoplasms.

In this study, we showed that downregulation of METTL14 contributes to autophagy inhibition in HSPCs, thereby significantly increasing their population. Notably, m6A is the most common RNA modification in eukaryotic cells, with the activity of the m6A-modified transcripts regulated by m6A RNA binding proteins. Notably, m6A modification influences mRNA alternative splicing, stability, translation, and subcellular localization. The m6A modification is a dynamic and reversible process, which is regulated by various protein complexes [41]. Research has shown that m6A modification is exerted by methyltransferases (Writers) [17], eliminated by demethylases (Erasers), and recognized by m6A binding proteins (Readers), which regulate m6A modification by altering the recognition of modified mRNA. The YTH domain protein family, along with proteins such as the IGF2BPs are important components of readers, whereby IGF2BPs can bind to m6A modification sites and enhance mRNA stability [33]. Previous studies have shown that m6A RNA methylation could alter the expression of essential autophagy-related (*Atg*) genes and play a crucial role in the regulation of autophagy [29]. Moreover, the effects of the m6A modification on autophagy are disease context-dependent [42]. For instance, m6A modification controls autophagy through upregulation of the ULK1 protein abundance [29]. Additionally, in some instance, the m6A modification directly inhibits autophagy [43], while in other cases, it promotes the initiation of autophagy [44]. Furthermore it can influence the formation of the autophagosomes to dysregulate autophagy [45]. Our findings further revealed that autophagy is significantly regulated by the METTL14, an observation we achieved by performing RNA-seq and MeRIP-seq analysis using the pathway enrichment analyses technique. We found that most of the autophagy genes were significantly decreased in Kras;M14^-/-^ HSPCs. Western blotting and Flow cytometry analysis results verified our hypothesis that silencing METTL14 caused autophagy dysfunction in HSPCs. Additionally, we demonstrated that METTL14 knockout reduces the stability and translation of mRNA by decreasing the m6A mRNA of autophagy-related genes and its binding to IGF2BP2, thereby inhibiting autophagy in Kras HSPCs. Autophagy is a highly regulated cellular process that involves the sequestration of proteins and damaged or senescent organelles into double-membrane vesicles known as autophagosomes. These autophagosomes subsequently fuse with lysosomes, resulting in the degradation and recycling of their contents. Autophagy dysregulation has been associated with the pathogenesis of several diseases, including neurodegenerative diseases, cardiomyopathy, infectious diseases, type II diabetes, fatty liver disease, and cancer. Autophagy plays a dual role in cancer: inhibiting benign tumor growth, while promoting advanced cancer growth [46]. The effect of autophagy on cancers is dependent on multiple factors, including tumor microenvironment, cancer type and stage, and genetic profile. numerous studies have shown that autophagy is the major contributor to the survival of cancer cells [47]. In this study, we demonstrated that the inhibition of autophagy reduces the excessive proliferation of *Kras*^*G12D/+*^ HSPCs. Additionally, the inhibition of autophagy inhibits both T-ALL and MPN development in *Kras*^*G12D/+*^ mice in a cell-autonomous manner. Consequently, we demonstrated that inhibition of autophagy significantly suppressed the progression of JMML.

Conclusively, our results demonstrate that inhibition of METTL14 effectively promotes the expansion of HSPCs and inhibits oncogenic Kras-induced leukemic disease process in vivo. Notably, the inhibition of the progression of JMML is significantly attributed to the inhibition of autophagy. Combined treatment of MEK and m6A inhibitors effectively suppressed the growth of mouse leukemia cells in vitro. This finding provided a robust rationale for developing therapies that target both pathways for the treatment of oncogenic Kras-driven myeloid malignancies.

## Materials and methods

### Mice

All mouse lines were maintained in a pure C57BL/6 genetic background (>N10). *Mettl14* conditional knockout mice (*Mettl14* ^*fl/fl*^; provided by Dr.Minghan Tong) were crossed to mice bearing a conditional oncogenic *Kras* (*Kras*^*Lox-stop-Lox (LSL) G12D/+*^) or *Mx1-Cre* mice to generate mice carrying both alleles (*Kras*^*LSL G12D/+*^; *Mettl14* ^*fl/fl*^and *Mettl14* ^*fl/fl*^*; Mx1- Cre*, respectively). *Kras*^*LSL G12D/+*^; *Mettl14* ^*fl/fl*^ mice were further crossed to *Mettl14* ^*fl/fl*^; *Mx1-Cre* mice to generate our experimental mice, including *Kras*^*LSL G12D/+*^;*Mettl14* ^*fl/fl*^ ;*Mx1-Cre*, *Kras*^*LSL G12D/+*^; *Mx1-Cre*. *Atg5* conditional knockout mice were crossed to mice bearing a conditional oncogenic *Kras* (*Kras*^*Lox-stop-Lox (LSL) G12D/+*^) or *Mx1-Cre* mice to generate mice carrying both alleles (*Kras*^*LSL G12D/+*^; *Atg5* ^*fl/fl*^ and *Atg5* ^*fl/fl*^; *Mx1- Cre*, respectively). *Kras*^*LSL G12D/+*^; *Atg5* ^*fl/fl*^ mice were further crossed to *Atg5* ^*fl/fl*^; *Mx1-Cre* mice to generate our experimental mice, including *Kras*^*LSL G12D/+*^;*Atg5* ^*fl/fl*^ ;*Mx1-Cre*,*Kras*^*LSL G12D/+*^; *Mx1-Cre*. CD45.1 positive congenic C57BL/6 recipient mice were purchased from Cyagen. All animal experiments were conducted in accordance with the *Guide for the Care and Use of Laboratory Animals* and approved by an Animal Care and Use Committee at Xi’an Jiaotong University(No.XJTUAE2020-2366). The program is accredited by the Association for Assessment and Accreditation of Laboratory Animal Care.

### Flow cytometric analysis of hematopoietic tissues

For PB samples, red blood cells were lysed in ammonium chloride solution (StemCell Technologies) prior to antibody staining. Cells isolated from BM and SP were resuspended in PBS with 2% fetal bovine serum (FBS) and passed through 25-μm cell strainers to obtain single-cell suspensions prior to antibody staining. Myeloid progenitors in BM and SP were analyzed as described in [48]. HSCs in BM and SP were analyzed as described by Kong et al [21]. The stained cells were analyzed on a FACS Calibur (BD Biosciences) or a CytoFLEX (Beckman Coulter Inc.). Antibodies specific for the following surface antigens were purchased from eBioscience: CD45.2 (104), CD45.1 (A20), B220 (RA3-6B2), CD19 (eBio1D3), Thy1.2 (53-2.1), Mac-1 (M1/70), Gr-1 (RB6-8C5), CD4 (GK1.5), CD8 (53-6.7), CD3 (145-2C11), IgM (II/41), IL7R (A7R34), Sca-1 (D7), TER119(TER-119), CD34 (RAM34), ckit (2B8), FcRII/III (93). CD150 (TC15-12F12.2) was purchased from BioLegend. HSCs were defined as Lin^-^CD48^-^ckit^+^Sac1^+^CD150^+^ cells, MPPs were defined as Lin^-^CD48^-^ckit^+^Sac1^+^CD150^-^ cells, MPs were defined as Lin^-^c-Kit^+^Sac1^-^ cells, CMPs were defined as Lin^-^c-Kit^+^Sac1^-^CD34^high^Fc^low^ cells, GMPs were defined as Lin^-^c-Kit^+^Sac1^-^CD34^high^Fc^high^ cells, MEPs were defined as Lin^-^c-Kit^+^Sac1^-^CD34^low^Fc^low^ cells and CLPs were defined as Lin^-^CD127^+^ckit^low^ Sac1^low^ cells. For detailed antibody description, see Table S1.

### Statistics

Three dependent biological replicates were performed in each experiment and values were presented as mean ± SD. Unpaired 2-tailed Student’s tests were used to determine the significance between two data sets, assuming significance at *P* < 0.05. Kaplan-Meier survival analysis was performed and survival differences between groups were assessed with the Log-rank test, assuming significance at *P* < 0.05. GraphPad Prism 8 were used for statistical analysis. A *p* value less than 0.05 was statistically significant.

Additional methods are described in Supplementary Materials and Methods.

## Supplementary information

supplementary_materials

Table S1

Table S2

Original Data File

## Data Availability

The accession numbers for the RNA-Seq and MeRIP-Seq data reported in this paper were deposited in GEO under the accession number GEO: GSE298134 and GSE298142. All data needed to evaluate the conclusions in the paper are present in the paper and/or the Supplementary Materials.
