# Supplementary material for: The m6A methyltransferase METTL14 promotes oncogenic Kras induced juvenile myelomonocytic leukemia through dysregulating autophagy
Source: Cell Death Differ. 2025 Aug 16;33(1):156–70. doi: 10.1038/s41418-025-01561-0 (PMC12811360; doi:10.1038/s41418-025-01561-0)
Supplement: Supplementary file 1 — supplementary_materials [file 41418_2025_1561_MOESM1_ESM.docx]

Supplementary Materials for

**The m6A methyltransferase METTL14 promotes oncogenic Kras induced juvenile myelomonocytic leukemia through dysregulating autophagy**

Peihua Zhang *et al.*

*Corresponding author. Guangyao Kong, [konggy@xjtu.edu.cn](mailto:konggy@xjtu.edu.cn)

Linlin Zhang, [zhanglinlin_fly@163.com](mailto:zhanglinlin_fly@163.com)

Zhanping Lu, [lu.006@163.com](mailto:lu.006@163.com)

**This PDF file includes:**

Supplementary Text

Figs. S1 to S6

Tables S1 to S2

Supplementary Materials and Methods

**Other Supplementary Materials for this manuscript include the following:**

Tables S1 to S2

**Supplementary Data**

Supplementary Table S1:Antibodies used in the study.

Supplementary Table S2: Primers in this study.


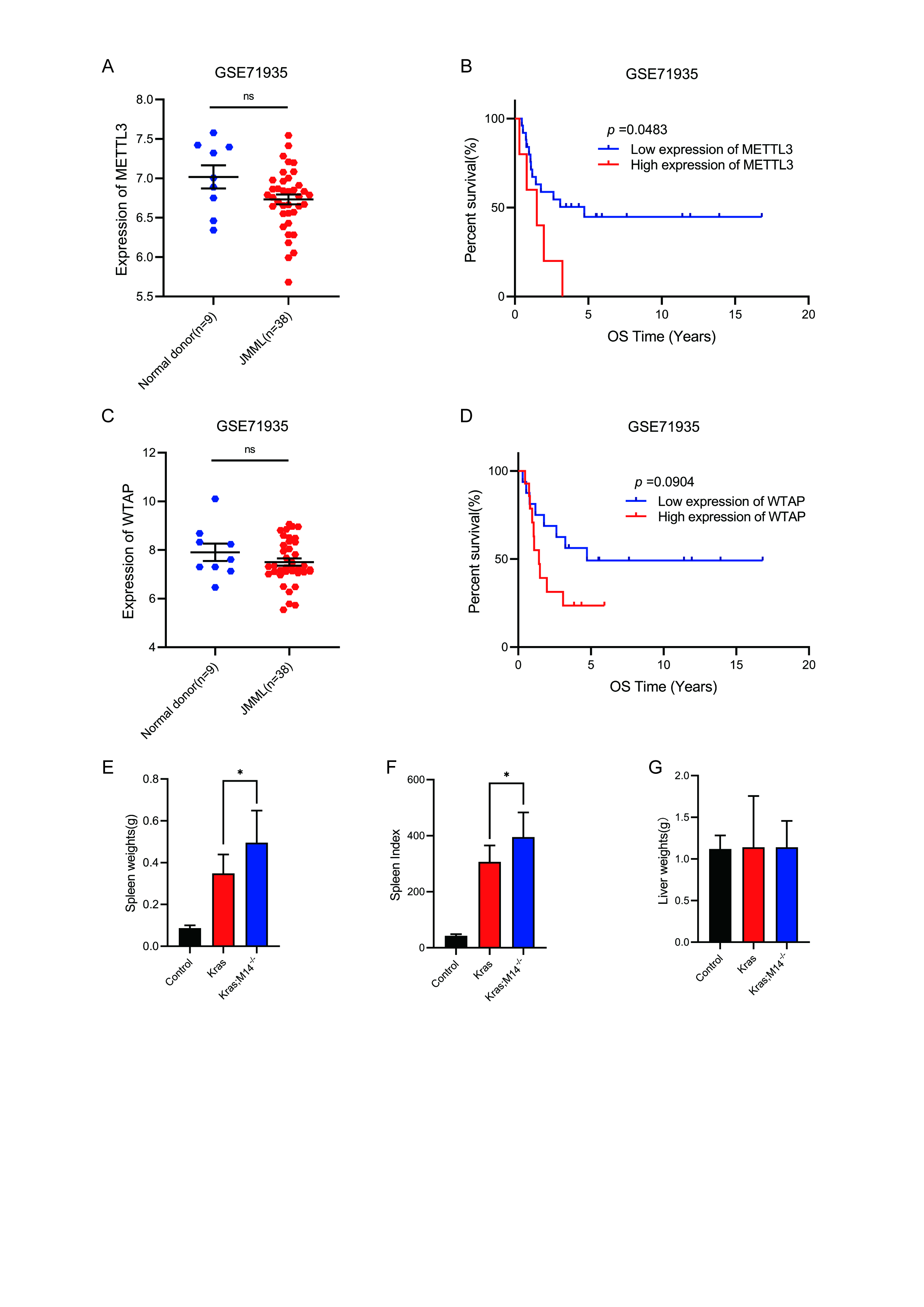


Fig.S1

**Fig.S1. High expression of METTL14 was associated with adverse outcomes in JMML patients, while its knockout significantly inhibited disease progression**

Control, Kras, and Kras;M14^-/-^ mice were sacrificed on day 1 of week seven for analysis of different hematopoietic tissues. (A) METTL3 expression in normal donors and JMML patients in GSE71935. (B) Kaplan-Meier plot. Survival curves for high (red) and low (blue) expression groups dichotomized at the optimal cutpoint are plotted and analysis of overall survival rate in the mutated cases of dataset GSE71935.(C) WTAP expression in normal donors and JMML patients in GSE71935. (D) Kaplan-Meier plot. Survival curves for high (red) and low (blue) expression groups dichotomized at the optimal cutpoint are plotted and analysis of overall survival rate in the mutated cases of dataset GSE71935. (E-G) Quantification of spleen weight (E) , spleen index (F) and liver weight(G). The results are presented as mean ± SD. * P<0.05; ** P<0.01; *** P<0.001.


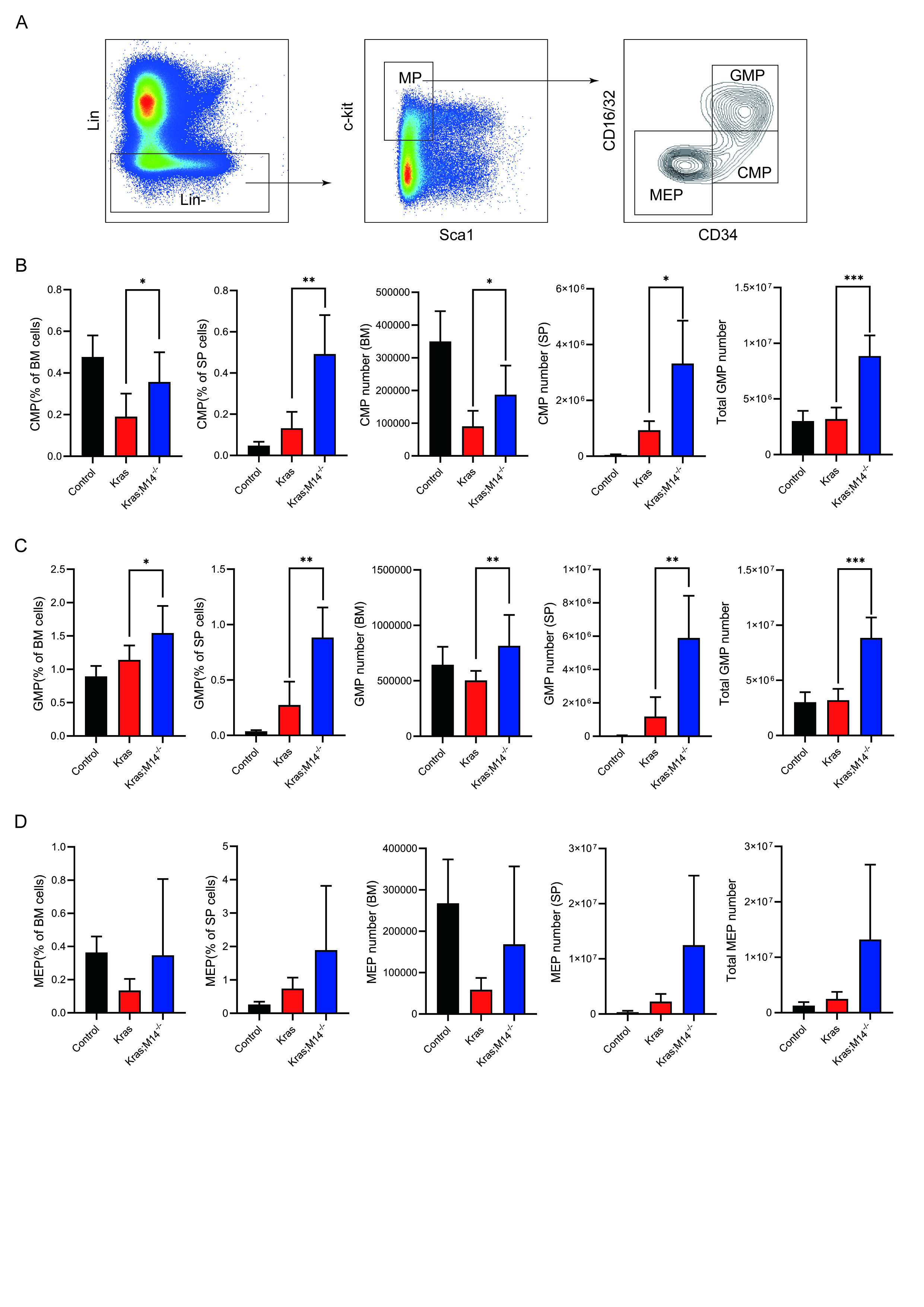


Fig.S2

**Fig.S2 Knockout of METTL14 rescues *Kras^G12D/+^*-mediated HSPCs depletion**

Control, Kras, and Kras;M14^-/-^ mice were sacrificed on day 1 of week seven for analysis of myeloid progenitor cells. (A) Representative example of staining and gating for MPs, CMPs, GMPs, and MEPs. (B-D) Quantitative analysis of CMPs (B), GMPs (C), and MEPs (D) in hind limb bone marrow (BM (H.L.)) and spleen (SP). The results are presented as mean ± SD. * P<0.05; ** P<0.01; *** P<0.001.


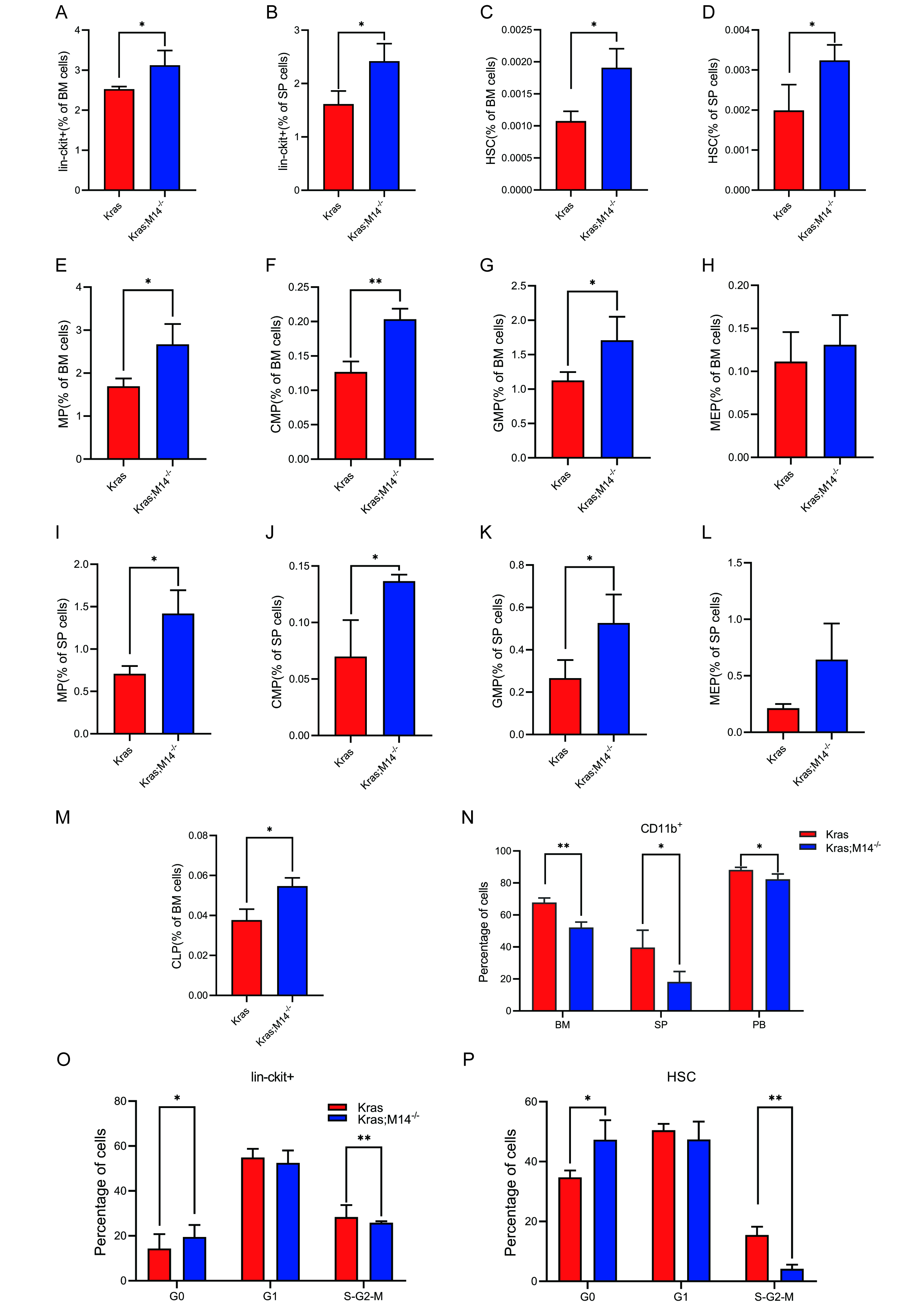


Fig.S3

**Fig.S3 The phenotypes of HSPCs in 8-week-old Kras and Kras;M14^-/-^ mice were detected**

Control, Kras, and Kras;M14^-/-^ mice were sacrificed on day 1 of week night for analysis of Lin^-^c-Kit^+^ (A-B), HSCs (C-D), MPs (E-L) and CLPs in hind limb BM (H.L.) and SP. (A-M) Quantitative analysis of Lin^-^c-Kit^+^ cells, HSCs, MPs, CMPs , GMPs, MEPs and CLPs in hind limb bone marrow (BM (H.L.)) and spleen (SP). (N) myeloid cell compartment in BM, SP and PB. (O-P) Cell cycle analysis of bone marrow Lin^-^c-Kit^+^ cells (O) and HSCs (P) using Ki67 and DAPI. The results are presented as mean ± SD. * P<0.05; ** P<0.01; *** P<0.001.


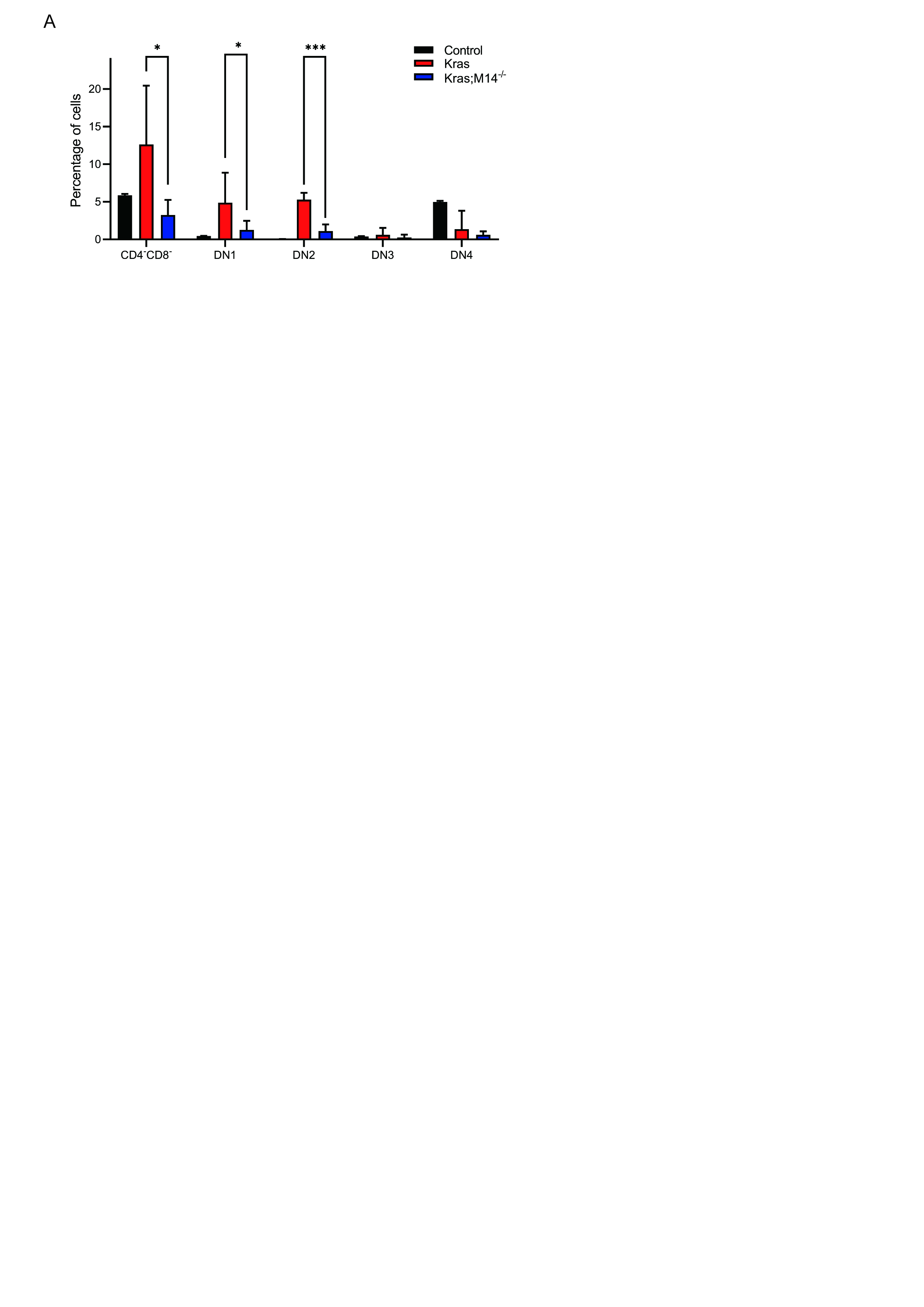


Fig.S4

**Fig.S4. Knockout of METTL14 suppresses oncogenic *Kras^G12D/+^*-induced myeloid disease in a cell-autonomous manner**

1. Quantitative analysis of donor-derived DN(CD4^-^CD8^-^) thymocyte, including DN1 (CD44^+^CD25^-^), DN2 (CD44^+^CD25^+^), DN3 (CD44^-^CD25^+^) and DN4(CD44^-^CD25^-^) thymocyte from moribund recipient mice. The results are presented as mean ± SD. * P<0.05; ** P<0.01; *** P<0.001.


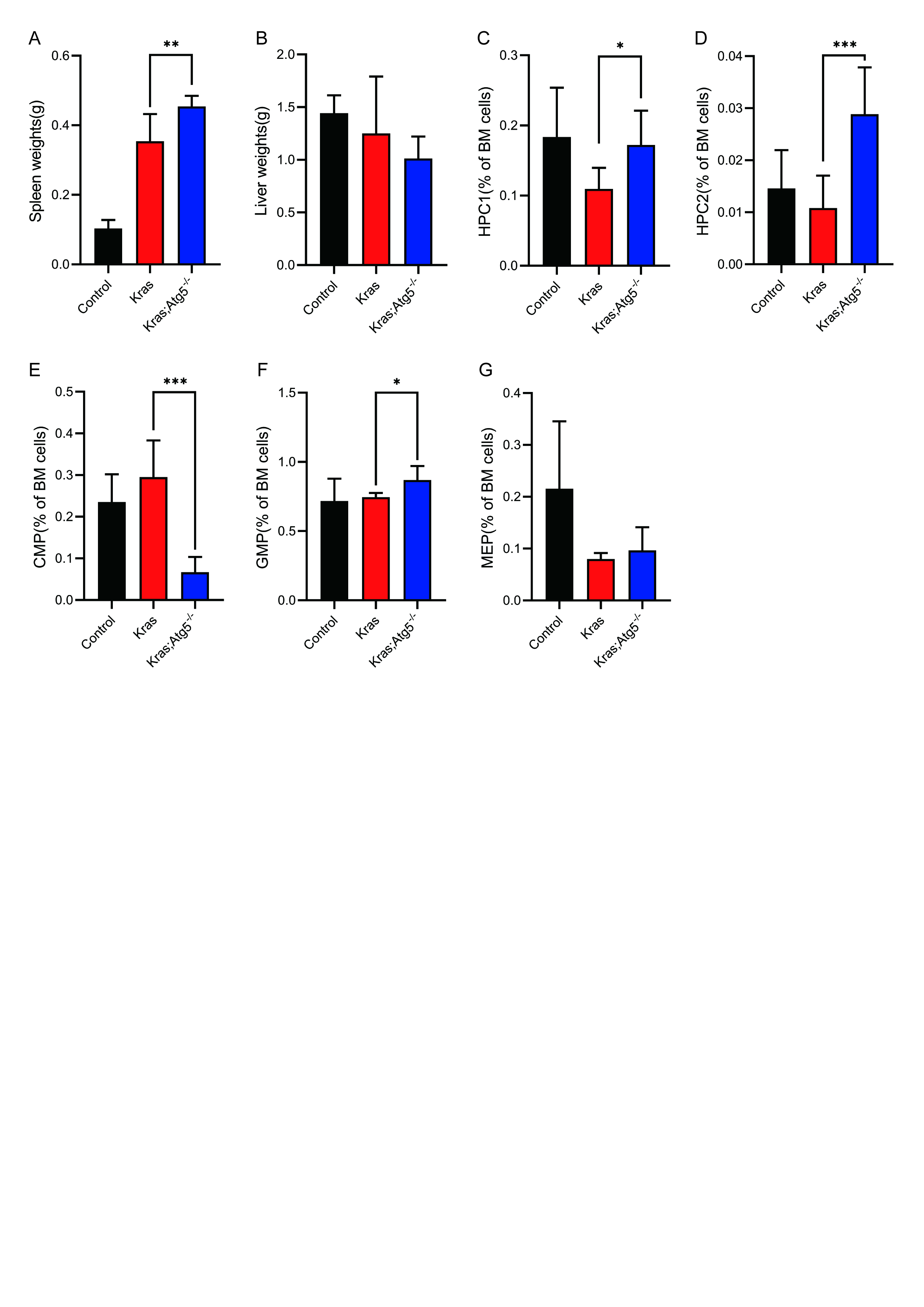


Fig.S5

**Fig.S5 Inhibition of autophagy via knockout of ATG5 promotes HSPCs amplification and suppresses the leukemic disease process.**

Control, Kras, and Kras;Atg5^-/-^ mice were sacrificed on day 1 of week seven for analysis of different hematopoietic tissues. (A-B) Quantification of spleen weight and liver weight. (C-G) Quantitative analysis of HPC1(C), HPC2(D), CMPs (E), GMPs (F), and MEPs (G) in hind limb bone marrow (BM (H.L.)). The results are presented as mean ± SD. * P<0.05; ** P<0.01; *** P<0.001.


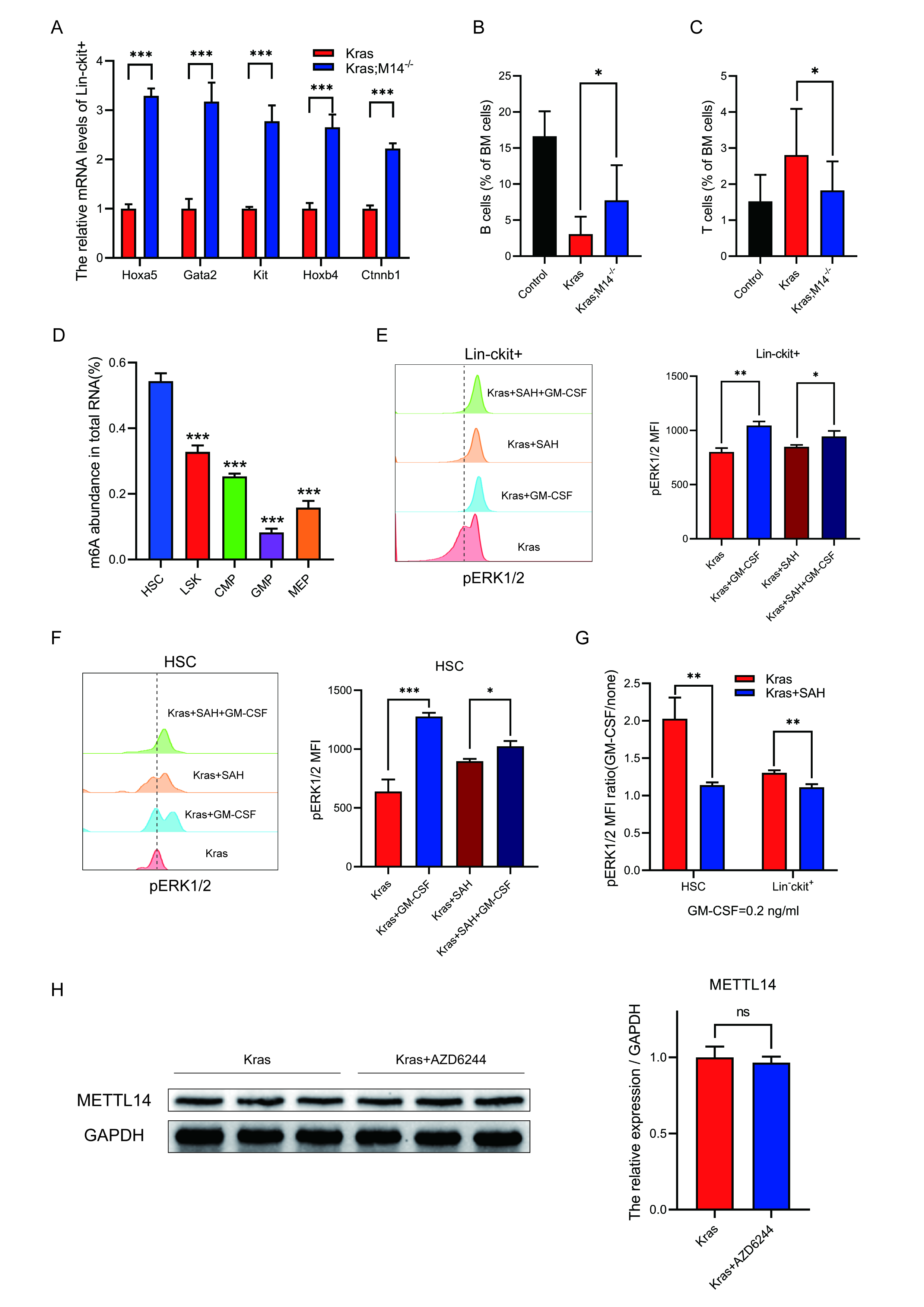


Fig.S6

**Fig.S6. The influence of METTL14 on HSPCs in the context of JMML.**

(A)RT-PCR showing mRNA levels of key regulators of HSC self-renewal in Kras and Kras;M14^-/-^ mice. (B) Frequency of B cells in bone marrow cells. (C) Frequency of T cells in bone marrow cells. (D) Quantification of total RNA m6A levels in HSPCs. (E-F) Whole bone marrow cells were sorted using flow cytometry and serum- and cytokine-starved for 2 hours at 37°C. Cells were stimulated with 32μM SAH and then stimulated with 0.2ng/ml of mGM-CSF for 10 minutes at 37°C. The level of p-ERK1/2 was measured using phospho-flow cytometry. Gating analysis was performed on Lin^−^cKit^+^ cells rich in myeloid progenitor cells and HSCs. (G) Quantification of median fluorescent intensity (MFI) of p-ERK1/2 in Lin^-^ckit^+^ cells and HSCs with and without mGM-CSF treatment. (H)Western blotting analysis of METTL14 protein levels in bone marrow cellswith and without AZD6244 treatment. The results are presented as mean ± SD. * P<0.05; ** P<0.01; *** P<0.001.

**Supplementary Materials and Methods**

**RNA m6A quantification**

Total RNA was extracted from bone marrow cells and HSPCs (Lin^-^ckit^+^ cells) using TRIzol® Reagent (Invitrogen, Germany). Then NanoDrop ND-1000 was used to determine the RNA purity and concentration. The m6A modification level of total RNA was examined via EpiQuik m6A RNA Methylation Quantification Kit (P-9005; Epigentek Group Inc., Farmingdale, NY, USA) according to the instruction. Briefly, 200 ng RNA accompanied with m6A standard were coated on assay wells, followed by capture antibody solution and detection antibody solution. The m6A levels were quantified colorimetrically by reading the absorbance of each well at a wavelength of 450 nm (OD450), and then calculations were performed based on the standard curve.

**Autophagy analysis**

Autophagy levels were measured by intracellular flow cytometry using the autophagomal marker LC3 protein and our cell surface panel for HSCs and progenitors. After RBC lysis, freshly harvested BM cells were starved in PBS on ice for 2 hours to induce autophagy by serum and cytokine starvation. In experiments inhibiting autophagy with chloroquine phosphate, cells were treated with autophagy inhibition reagent for 4 h. After staining with lineage and cell surface antibodies as described above, the cells were stained, fixed, and permeabilized using the Cytofix and Cytoperm solutions from the BD Pharmingen™ Transcription Factor Buffer Set (BD Pharmingen™, BD562574) according to the manufacturer’s protocol. The stained, fixed, and permeabilized cells were resuspended in Perm/Wash buffer containing the LC3 antibody. Cells were washed with Perm/Wash buffer to remove residual unbound antibody and resuspended in fresh Perm/Wash buffer followed by flow cytometry analysis on a CytoFLEX (Beckman Coulter Inc.). Analysis of all flow cytometry data was performed using FlowJo software (V10). For detailed antibody description, see table S1.

**Colony forming analysis**

The methylcellulose based medium M3234 were obtained from StemCell Technologies. To detect colony forming unit, 5×10^4^ bone marrow cells isolated from various groups of mice were plated in duplicate in M3234 in the absence or presence of various concentrations of GM-CSF according to the manufacturer’s protocol. Colonies were counted after 7 days in culture and same numbers of cells were re-plated.

**Transplantation experiments**

For bone marrow cell transplantation described in Figure5, 2.5×10^5^ bone marrow cells along with 2.5×10^5^ competitor bone marrow cells (CD45.1+) were injected into lethally irradiated CD45.1+ recipients as previously described(1). Four weeks after transplantation, Cre expression was induced through intraperitoneal injection of 5µg/g body weight of polyinosinic-polycytidylic acid (pI-pC, Sigma) every other day twice. FACS analysis of peripheral blood was performed at 4-week intervals after transplantation. Donor-derived hematopoiesis was analyzed as previously described(2).

**Complete blood count (CBC)**

CBC analysis was performed using a Hemavet 950FS (Drew Scientific).

**Genotyping of leukemia cells**

Genomic DNAs were extracted from bone marrow and Spleen of diseased recipient mice using Mouse Direct PCR Kit (For Genotyping) (Selleck). Genotyping of the floxed or recombined *Kras^G12D/+^* and *Mettl14*/*Atg5* alleles was performed as previously described(3). For detailed primers description, see table S2.

**Cell cycle analysis**

Cell cycle analysis was performed essentially as previously described(4). Briefly, bone marrow cells were labeled with following antibodies for myeloid progenitor cells: FITC-B220 (RA3-6B2), FITC-CD3 (145-2C11), FITC-CD4 (GK1.5), FITC-CD8 (53-6.7), FITC-Gr-1 (RB6-8C5), FITC-CD19 (eBio1D3), FITC-IgM and FITC-TER-119 (TER-119), PerCP Cy5.5-Sca1 (D7), APC-CD48, APC-eFluro 780-c-Kit (2B8), PE-Cy7-CD150. Stained cells were fixed in 2% Paraformaldehyde (Electron Microscopy Sciences)/PBS, and permeablized in 0.1% Saponin (Sigma)/PBS containing PE-Ki67 (BD Biosciences) and DAPI (Sigma). All of the antibodies were purchased from eBioscience unless specified. The stained cells were analyzed on a CytoFLEX (Beckman Coulter Inc.).

**Methylated RNA immunoprecipitation sequencing (MeRIP-seq) and RNA sequencing (RNA-seq)**

Total RNA was extracted from the HSPCs (Lin^-^ckit^+^ cells) sorted by magnetic beads using TRIzol® Reagent (Invitrogen, Germany). The RNA integrity was assessed by Bioanalyzer 2100 (Agilent, CA, USA). Dynabeads Oligo (dT)25-61005 (Thermo Fisher, CA, USA) was used to capture Poly (A) RNA. Then the Poly (A) RNA was incised into pieces under 86 ° C for 7 minutes by Magnesium RNA Fragmentation Module (NEB, cat. e6150, USA). In the IP buffer (50 mM Tris-HCl, 750 mM NaCl and 0.5% Igepal CA-630), the cleaved RNA fragments and m6A Antibody (No. 202003, Synaptic Systems, Germany) were premixed and IP. Then DNA was synthesized, and the compound duplex of DNA and RNA was converted into DNA duplex. Next, dUTP (Thermo Fisher, article No. R0133, USA) was incorporated into the duplex. An A base was added to the end of the double-stranded DNA, and the fragment size was screened and purified using magnetic beads (insert size: 180-220bp). UDG enzyme (NEB, cat.m0280, USA) was used to digest the two strands, and then PCR was performed under the following conditions to form a sequencing library: initial denaturation at 95℃ for 3 min; 8 cycles of denaturation at 98℃ for 15 sec, annealing at 60℃ for 15 sec, and extension at 72℃ for 30 sec; and then final extension at 72℃ for 5 min. At last, Illumina Novaseq™ 6000 (LC-Bio Technology CO., Ltd., Hangzhou, China) was used for double-ended sequencing with PE150 sequencing mode following the standard protocol.

For RNA sequencing, total RNA was extracted from the HSPCs (Lin^-^c-Kit^+^ cells) sorted by magnetic beads using TRIzol® Reagent (Invitrogen, Germany) following the protocol as described previously. Other processes were performed by Omic Studio technologies (Hangzhou, China) and Oebiotech (Shanghai, China).

**Quantitative real-time polymerase chain reaction (qRT-PCR)**

Total RNA was extracted from the HSPCs (Lin^-^ckit^+^ cells) sorted by magnetic beads using TRIzol® Reagent (Invitrogen, Germany). Then NanoDrop ND-1000 was used to determine the RNA purity and concentration. The cDNA was synthesized from total RNA using a Primescript RT reagent kit (Takara). Then, qRT-PCR was performed by SYBR Premix Ex Taq™ II (CWBIO, China). The reaction conditions were as follows: 95 °C for 30 s, 40 cycles of 95 °C for 5 s, and 60 °C for 30 s. The above experimental steps were operated in accordance with the manufacturer's protocols. Primers were synthesized by Tsingke (Beijing, China), and the sequences used are listed in table S2. The 2^-ΔΔCT^ method was used to calculate the gene expression level.

**Western blotting**

Cell lysate samples were collected in RIPA buffer (POINEER Biotechnology, Xi'an, China), including a protease inhibitor cocktail (NCM Biotech, Suzhou, Xi'an). About 60μg protein lysates were separated with 10% SDS-polyacrylamide gels and transfected to PVDF membranes. Then the membranes were blocked into 5% BSA/PBST for 1h at room temperature and incubated with primary antibodies at 4°C overnight. Next, the membranes were incubated with secondary antibodies for 1h at room temperature. Finally, the protein bands were visualized with chemiluminescence reagents (ShareBio, Shanghai, China). The antibodies were used in this study as follows: METTL14 (cat. no. 80790-1-RR; Proteintech), LC3(cat. no. 12741S; Cell Signaling Technology, CST), GAPDH (cat. no. 2118S; CST), P62(cat. no. 18420-1-AP; Proteintech).

**MeRIP-qPCR**

MeRIP assay was performed by the BersinBioTM MeRIP Kit (BersinBio, Guangzhou, China) according to the manufacturer’s instruction. Firstly, we used TRIzol regent to isolate total RNA as described previously. Then the RNA was segmented into about 300-nucleotide fragments. Next, the fragments were incubated with 5μg anti-IgG or anti-m6A (68055-1-Ig, Proteintech) for 2 h at 4 ℃. Moreover, we mixed Protein A/G magnetic beads and antibody-treated RNA in IP buffer for 2 h at 4 ℃. The RNA was washed, then eluted with Proteinase K and elution buffer for 1 h at 55 ° C. At last, the RNA was purified with mixture of phenol: chloroform: Isoamyl Alcohol (25:24:1). The methylated RNA expression was measured by qRT-PCR.

**RNA immunoprecipitation (RIP) assay**

RIP-qPCR was performed using the BersinBioTM RNA Immunoprecipitation Kit (BersinBio, Guangzhou, China) according to the manufacturer’s instruction. Cell lysates were divided into 3 groups, anti-IgG, anti-IGF2BP2, and input. Then Polysome lysis buffer was used to lyse cells and the cell lysate was divided into anti-IGF2BP2, anti-IgG (1mg/ml, Cell Signaling Technology) and input samples. Then, 5μg specific antibodies of anti-IgG or anti-IGF2BP2, protein A/G magnetic beads and cell lysates were incubated at 4 ℃ overnight. The lysates were washed, then digested with Proteinase K for 1 h at 55 ° C. At last, the RNA was purified with mixture of phenol: chloroform: Isoamyl Alcohol (25:24:1). Target RNA levels were detected by qRT-PCR.

**RNA decay assay**

Bone marrow cells in Kras and Kras;M14^-/-^ mice were treated with Actinomycin D (5μg/ml, MCE, USA) for 0,1,2,4, 6, and 8h. Then total RNA was isolated, and qRT-PCR was conducted as described previously. 18s rRNA was used as an internal control. The RNA lifetime (t_1/2_) was calculated using the equations below: Nt/N0 = e ^– Kdecay*t^, t_1/2_ = ln2/K_decay_.

**Cell cultures**

Cell cytotoxicity was detected by CCK8 assays. Bone marrow cells from moribund Kras mice were cultured in IMDM with 10% FBS, 0.2ng/ml mGM-CSF. Cells were seeded at 2 X 10^5^/ml in triplicate in 96-well plates in the presence of DMSO or various concentrations of AZD6244 and/or SAH. After 5 days (Kras cells) in culture, cell viability was determined using the absorbance at 450 nm by a microplate reader, and 10 μl of CCK8 solution was added to each well 2 hours in advance. The halfmaximal inhibitory concentration (IC50) and Combination Index (CI) were calculated using Compusyn software. A CI value < 1 indicated synergism.

**References**

1. Kong G, Chang YI, Damnernsawad A, You X, Du J, Ranheim EA, et al. Loss of wild-type Kras promotes activation of all Ras isoforms in oncogenic Kras-induced leukemogenesis. Leukemia. 2016;30(7):1542-51.

2. Damnernsawad A, Kong G, Wen Z, Liu Y, Rajagopalan A, You X, et al. Kras is Required for Adult Hematopoiesis. Stem Cells. 2016;34(7):1859-71.

3. Tuveson DA, Shaw AT, Willis NA, Silver DP, Jackson EL, Chang S, et al. Endogenous oncogenic K-ras(G12D) stimulates proliferation and widespread neoplastic and developmental defects. Cancer Cell. 2004;5(4):375-87.

4. Kong G, Wunderlich M, Yang D, Ranheim EA, Young KH, Wang J, et al. Combined MEK and JAK inhibition abrogates murine myeloproliferative neoplasm. J Clin Invest. 2014;124(6):2762-73.
