# Supplementary figures and images for: The m6A methyltransferase METTL14 promotes oncogenic Kras induced juvenile myelomonocytic leukemia through dysregulating autophagy
Source: Cell Death Differ. 2025 Aug 16;33(1):156–70. doi: 10.1038/s41418-025-01561-0 (PMC12811360; doi:10.1038/s41418-025-01561-0)

**Figure 1**

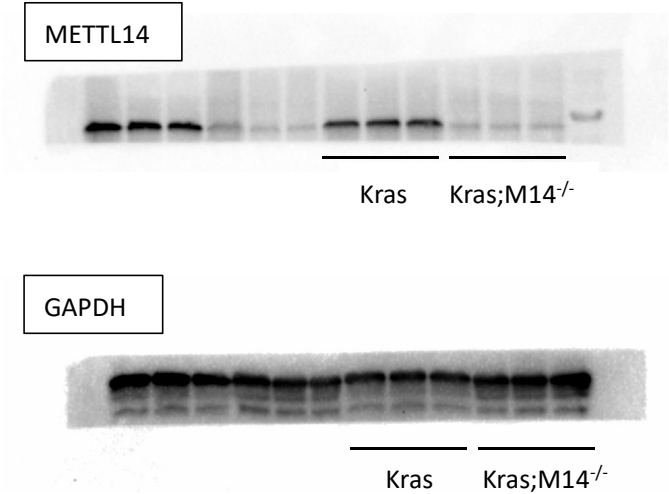

**Figure 6**

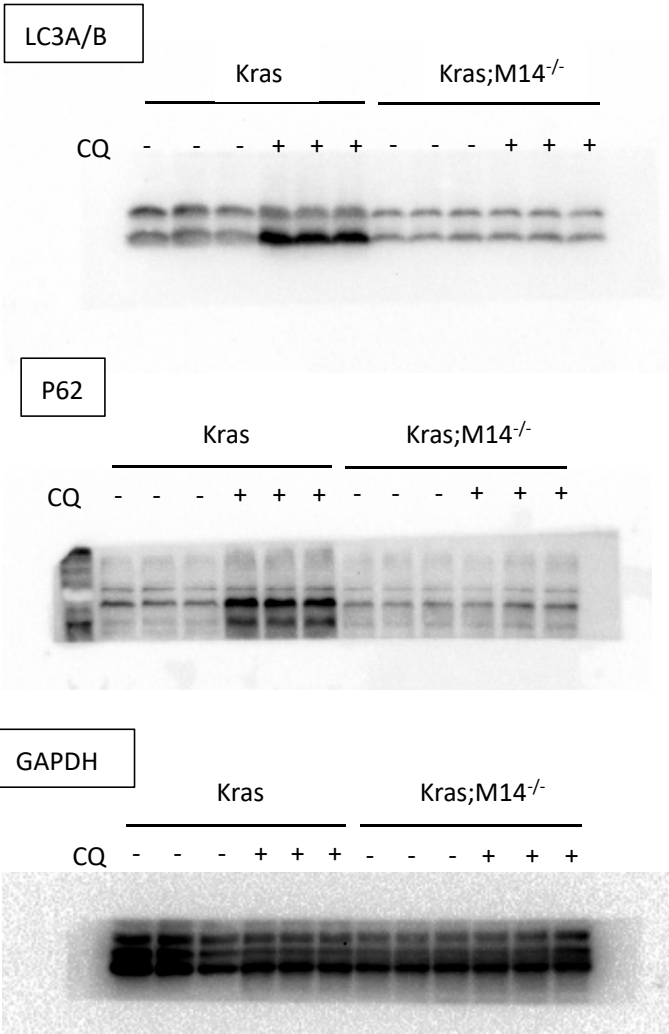

**Figure S5**

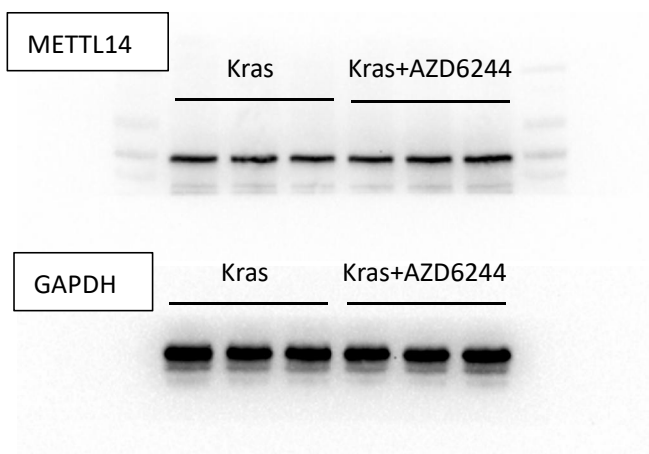

Supplement: Supplementary file 4 — Original Data File [file 41418_2025_1561_MOESM4_ESM.pdf]
